# Expression Profiling of Four Mango FT/TFL1-Encoding Genes under Different Fruit Load Conditions, and Their Involvement in Flowering Regulation

**DOI:** 10.3390/plants11182409

**Published:** 2022-09-15

**Authors:** Itamar Gafni, Avinash Chandra Rai, Eyal Halon, Tali Zviran, Isaac Sisai, Alon Samach, Vered Irihimovitch

**Affiliations:** 1 Institute of Plant Sciences, Agricultural Research Organization (ARO), The Volcani Center, Rishon LeZion 7528809, Israel; 2 The Robert H. Smith Institute of Plant Sciences and Genetics in Agriculture, Faculty of Agriculture, Food and Environment, The Hebrew University of Jerusalem, Rehovot 76100, Israel

**Keywords:** alternate bearing, flowering, FLOWERING LOCUS T (FT), fruit load, mango, TERMINAL FLOWER 1 (TFL1)

## Abstract

Plant flowering is antagonistically modulated by similar FLOWERING LOCUS T (FT) and TERMINAL FLOWER 1 (TFL1) proteins. In mango (*Mangifera indica* L.), flowering is induced by cold temperatures, unless the tree is juvenile or the adult tree had a high fruit load (HFL) in the summer. Here, we studied the effects of juvenility and fruit load on the expression of four *MiFT*/*TFL1* genes cloned from the mango ‘Shelly’ cultivar. Ectopic expression of *MiFT1* in *Arabidopsis* resulted in early flowering, whereas over-expression of *MiFT2* and the two cloned *MiTFL1* genes repressed flowering. Moreover, juvenility was positively correlated with higher transcript levels of *MiFT2* and both *MiTFL1*s. In trees with a low fruit load, leaf *MiFT1* expression increased in winter, whereas HFL delayed its upregulation. *MiFT2* expression was upregulated in both leaves and buds under both fruit load conditions. Downregulation of both *MITFL1*s in buds was associated with a decrease in regional temperatures under both conditions; nevertheless, HFL delayed the decrease in their accumulation. Our results suggest that cold temperature has opposite effects on the expression of *MiFT1* and the *MiTFL1*s, thereby inducing flowering, whereas HFL represses flowering by both suppressing *MiFT1* upregulation and delaying *MiTFL1s* downregulation. The apparent flowering-inhibitory functions of *MiFT2* are discussed.

## 1. Introduction

Mango (*Mangifera indica* L.), grown in tropical and subtropical climate zones, is an important fruit tree crop [1,2]. Despite the fact that global demand for mango is rapidly increasing [3], its production is frequently limited. In mango, flower induction occurs in early winter; anthesis, fertilization and fruit set in early spring, and further fruit development occurs during the late spring and summer [4,5]. Environmental conditions that interfere with any of these essential processes might reduce fruit yield. In addition, high fruit load (HFL) during the summer can inhibit flower induction in the following winter [4]. The negative effect of HFL on the following year’s flowering is observed in various tree species, causing alternate bearing (AB) or biennial fruit bearing [6,7,8]. In some mango cultivars, it is not clear whether the loss of yield after a high load crop year is due to reduced flowering or to other events occurring after flower induction [1,2,6,7,8].

When grown from seeds, the juvenile phase in mango lasts approximately 3 years, followed by an adult reproductive phase, in which flowering and fruiting take place [1]. Typically, mango inflorescences are born from terminal buds of mature new shoots, and initiation of new shoots is therefore the first event required for flowering [5]. Several studies have shown that under subtropical conditions, most mango cultivars flower only after exposure to cool temperatures, namely, after at least 1 month of day and night temperatures of 13–19 °C; higher temperatures (above 20 °C) inhibit flowering [9,10,11]. Moreover, because the presence of mature leaves on shoots is a mandatory prerequisite for flowering, it has been suggested that under floral-inductive cool temperatures, a floral-promoting signal is generated in the leaves of adult trees, and is transferred to the shoot apical meristem to promote floral transition [5]. Other factors are also implicated in affecting mango flowering. For instance, based on studies showing that exogenous application of gibberellins (GAs) enhances mango vegetative growth and represses flowering [12,13,14], it has been proposed that in mango, similar to other fruit crop tree species, GAs inhibit flowering [7]. In line with this prediction, paclobutrazol—a plant growth regulator that inhibits GA synthesis—can be applied to stimulate mango flowering in warm tropical growing zones, or to induce off-season flowering in subtropical zones [14,15,16].

The transition from vegetative to reproductive phase is an important developmental process in flowering plants. It might occur in response to external factors (such as temperature changes), endogenous signals (such as age, changes in sugar or hormone availability), or both. Studies in *Arabidopsis* have shown that the transition to flowering is antagonistically modulated by two closely related conserved proteins that display structural similarities to the mammalian phosphatidylethanolamine-binding protein (PEBP): FLOWERING LOCUS T (FT) and TERMINAL FLOWER 1 (TFL1) [17,18,19]. While FT encodes the florigen protein, which is transported via the phloem from the leaves to the shoot meristem, where it triggers the initiation of floral transition, *TFL1* acts in the meristem as a local repressor that maintains the indeterminate identity of the shoot meristem [20,21,22,23]. Moreover, in *Arabidopsis*, the PEBP-like family is comprised of four additional members: *TWIN SISTER OF FT* (*TSF*), *MOTHER OF FT* (*MFT*)*, ARABIDOPSIS THALIANA CENTRORADIALIS HOMOLOGUES* (*ATC*), and *BROTHER OF FT* (*BFT*). In particular, whereas *TSF* and *MFT* function redundantly with *FT* to promote flowering [24,25]*, ATC* and *BFT* assume floral-inhibitory roles, similar to *TFL1* [26,27]. The modus operandi of FT/TFL1, leading to their antagonistic functions, is complex. Briefly, in the shoot meristem, FT associates with the basic leucine zipper transcription factor FD and 14-3-3 proteins to form a ‘floral-activation complex’. This complex activates the expression of MADS box genes, such as *APETALA1* (*AP1*) and *SUPPRESSOR OF OVEREXPRESSION OF CONSTANS1* (*SOC1*), leading to flowering evocation [22,28]. TFL1, on the other hand, is suggested to act as a floral repressor, competing with FT for binding to FD and 14-3-3 proteins, thus leading to the formation of a ‘floral-repressor complex’ that suppresses the floral transition [29,30,31]. Indeed, in line with this proposed model, a recent study showed that the antagonism between FT and TFL1 relies on their competition for the chromatin-bound FD at different shared target loci [32]. Furthermore, the opposite functions of FT and TFL1 proteins were mapped to two conserved amino acids that have been reported as key to promoting flowering, as well as to a short conserved amino acid sequence (termed B-segment) found in the C-terminal region of FT [33,34]. Other key amino acids, including those necessary for the 14-3-3 interaction and FT movement, were also identified [28,35].

Comparative and functional studies have proven the conservation of *FT/TFL1* flowering regulators in diverse plant species (reviewed by [18,19,30,31]). In addition, the existence of multiple copies of *FT-like* and *TFL1-like* genes has been reported in different plant species. Interestingly, it has also been reported that during evolution, some *FT* homologs seem to have evolved to function as flowering repressors [18,30,31]. In sugar beet, two *FT-like* genes exist*,* whereas *BvFT1* represses flowering, *BvFT2* promotes it [17]. Similarly, in *Nicotiana tabacum*, which possesses five *FT-like* genes, three of them (*NtFT1-3*) are suggested to act as floral inhibitors, and the other two (*NtFT4* and *NtFT5*) as floral inducers [36,37]. In both cases, the *FT*-like genes that assume repressor functions display a semi-conserved B-segment, with distinct non-conserved amino acid substitutions in the segment extending from amino acid positions 128–141 (corresponding to *At*FT) [17,37]. Moreover, characterization of *FT/TFL1* genes in different species has revealed a wider impact on plant development than originally thought. For instance, besides flowering control, FT-like proteins are suggested to play roles in controlling stomatal opening and seed germination, whereas TFL1-like proteins are suggested to take part in the control of juvenility, and in affecting shoot growth patterns [18,30,31].

Homologs of *Arabidopsis FT/TFL1* genes have been identified in various fruit tree species. Moreover, in some cases, functional evidence has been presented of these proteins affecting flowering (reviewed in [7,8]). Indeed, early flowering was shown to be induced by overexpression of *FT-like* genes in citrus [38], apple [39], olive [40], and kiwifruit [41], whereas an early flowering phenotype could also be achieved by reduced expression of *TFL1-like* gene transcripts, as shown, for example, in pear [42] and apple [43]. Furthermore, studies of different commercial AB cultivars of citrus [44,45,46], avocado [47], olive [40] and apple [48] fruits showed that the presence of fruit on the tree affected the expression of different flowering-related genes in both leaves and buds.

In mango, several studies have led to the identification of distinct *FT/TFL1* family members. Nakagawa et al. [4] were the first to report on the isolation of a *FT-like* gene from mango cv. Irwin, showing that its expression increased during the winter in leaves of de-fruited trees which flowered intensively in the spring, whereas HFL and GA_3_ treatment repressed its expression. Further studies using mango cvs. Alphonso and SiJiMi led to the isolation of three *FT-like* genes (*MiFT1–3*) [49,50]. Sequence analysis indicated that *MiFT1* and *MiFT2* from ‘Alphonso’ corresponded to *MiFT1* and *MiFT2* from ‘SiJiMi’. Moreover, *MiFT1* of both cultivars corresponded to the *MiFT* sequence in ‘Irwin’ [49], whereas the sequences of *MiFT3* differed between the two cultivars [49,50]. Interestingly, based on expression analysis results, it was suggested that the *Mi*FTs’ functions might differ between the cultivars [50]. Finally, ectopic expression of the three isolated *MiFT* genes from ‘SiJiMi’ in *Arabidopsis* resulted in early flowering, but *MiFT1* displayed the strongest effect [50]. Regarding the *MiTFL1* genes, Wang et al. [51] recently reported on the isolation of four *TFL1-like* genes (*MiTFL1-1–4*) from cv. SiJiMi. Based on findings showing that overexpression of the four ‘SiJiMi’ *MiTFL1* genes in *Arabidopsis* delays flowering time, it was suggested that all *MiTFL1*s play roles in regulating mango flowering [51]. Further details on their specific modes of action remain to be established. The current release of different mango genome drafts [52,53], opens now new possibilities to explore the mango PEBP-like gene family. As such, the ‘Tommy Atkins’ genome assembly includes approximately  86% of the  439 Mb haploid mango genome [52].

Mango ‘Shelly’ cultivar was developed by the Israeli breeding project and has been planted extensively since the early 2000s in commercial orchards throughout Israel [54]. It was bred as a late-ripening cultivar, with harvest season starting in September to mid-November; nevertheless, growers in the north of Israel tend to harvest it earlier, even in late August. Moreover, despite its increasing popularity, there are controversial reports on its tendency toward AB behavior. Here, we aimed to determine if and how HFL affects ‘Shelly’ flowering, and to elucidate the possible roles of distinct ‘Shelly’ *FT/TFL1* genes in modulating its flowering. We first performed de-fruiting treatments to help define the nature of the AB in ‘Shelly’. We next isolated four ‘Shelly’ *FT/TFL1* genes and assessed their functions in flowering regulation by ectopic expression in *Arabidopsis*. We also monitored their expression levels in tissues of juvenile and adult trees, and in leaves and terminal buds of trees under different fruit load conditions. The information provided herein will serve to improve our understanding of the AB behavior of the ‘Shelly’ cultivar, and of the potentially distinct functions of *MiFT/TFL1* family members. We compared our findings to previous results in other mango cultivars, and discuss the identified differences. The possible mechanisms by which changes imposed by ambient temperature, together with the **“**memory**”** of HFL on the tree, affect mango flowering are discussed.

## 2. Results

### 2.1. Exploring the Effects of Crop Load on Return Flowering in Mango ‘Shelly’ Cultivar

**Figure 1:**
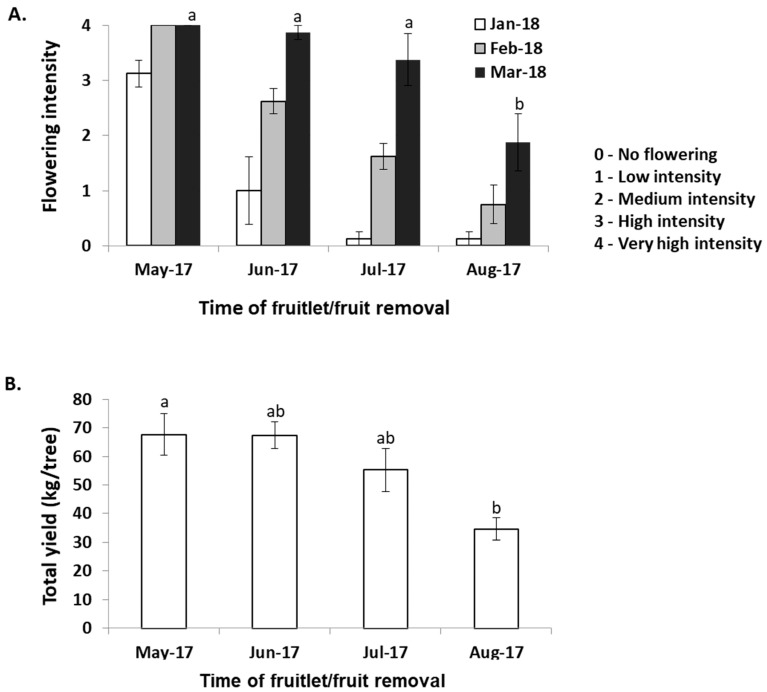
Effects of fruit load on flowering and yield in the next season. (**A**) Effects of de-fruiting treatments on return to flowering. Complete fruit-removal treatments, conducted from May 2017 through August 2017, were performed at the beginning of May, June and July, and at the end of August (representing the commercial harvesting period). Flowering intensity levels of individual trees within each group were ranked at the beginning of January and February and at the end of March 2018, using the following scale: (0) no flowering; (1) low intensity—<20 inflorescences; (2) medium intensity—<50 inflorescences; (3) high intensity—<150 inflorescences; (4) very high intensity—>150 inflorescences. (**B**) Effects of de-fruiting treatments on yield in the next season. Total yield per tree was determined in the following commercial harvest season (August 2018). Values represent means ± SE of four trees per treatment, and different letters indicate significant differences according to Tukey–Kramer multiple comparison test, with *p* ≤ 0.05.

Under our local growing conditions, in northern Israel, the ‘Shelly’ cultivar blooms in the spring, with peak flowering usually occurring in March to April, although inflorescences can already be detected in January. To examine whether and to what degree HFL in ‘Shelly’ represses return to flowering, we selected uniform heavily producing trees and performed manual de-fruiting treatments at different time points (see Section 4.1). Flowering records, collected from January through to March, demonstrated that heavy fruit loaded trees, whose fruits remained on the trees until August (at the time of commercial harvest), displayed the lowest levels of return to flowering. The fruit removal/harvest at this time point resulted in a significantly lower flowering score than for the trees in the other groups, which were de-fruited earlier (see Figure 1A). Fruit removal in the HFL trees at earlier stages had no significant effect on the flowering magnitude. In this case, the flowering scores recorded in March, for trees which had been de-fruited in June and July, were almost the same as those trees which had been de-fruited at the beginning of May (mimicking natural *off* conditions). Nevertheless, the results also showed that when the de-fruiting treatments were delayed, so was the inflorescence appearance (Figure 1A). Lastly, determination of the following season’s total yields demonstrated a direct correlation between the recorded flowering scores and the following year’s yields (Figure 1B). Taken together, the results indicated that in ‘Shelly’, HFL suppresses, but does not completely abolish the next season’s flowering.

### 2.2. Identification and Characterization of MiFT/MiTFL1 Transcripts from Mango ‘Shelly’ Cultivar

As mentioned in Section 1, several studies with different mango cultivars have led to the identification of distinct mango *FT/TFL1* family members [4,49,50,51]. To obtain cDNAs of *MiFTs* and *MiTFL1s* from ‘Shelly’, we used primers designed within the 5′ and 3′ untranslated regions (UTRs) of distinct *MiFTs* and *MiTFL1s* from cultivars with annotated sequences (see Section 4.2). The PCR products were cloned, resulting in the isolation of two full-length sequences of *FT* encoding 181- and 197-amino acid proteins (which we named *MiFT1* and *MiFT2*, respectively), and two full-length sequences of *TFL1* encoding 171- and 174-amino acid proteins (*MiTFL1-1* and *MiTFL1-2*, respectively).

**Figure 2:**
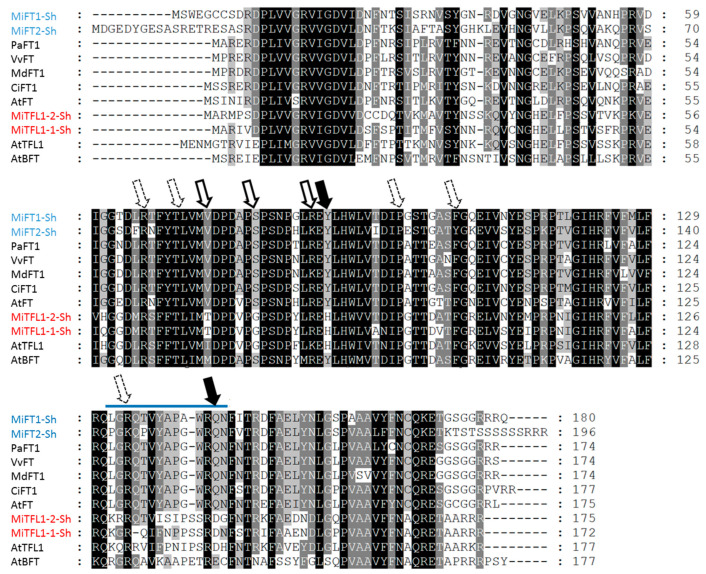
Amino acid sequence comparison and structural features of mango ‘Shelly’ cultivar MiFT1, MiFT2, MiTFL1-1 and MiTFL1-2. Comparison of the deduced amino acid sequences of the four *MiFT/TFL1* genes isolated from ‘Shelly’ with representative FT and TFL1 proteins from: avocado (*Persea americana*) *Pa*FT (accession no. [ac]: AIG92770.1), grape (*Vitis vinifera*) *Vv*FT1 (ac: ABL98120.1), citrus (*Citrus unshiu*) *Ci*FT1 (ac: BAA77836.1), apple (*Malus x domestica*) *Md*FT (ac: ACL98164.1) and *Arabidopsis* (*Arabidopsis thaliana*) *At*FT (ac: BAA77838.1); and with BFT and TFL1 proteins from *Arabidopsis*, *At*TFL1 (ac: AED90661.1) and *At*BFT (ac: Q9FIT4.1). Residue shading in black, dark gray and light gray indicates 100%, 75%, and 50% amino acid similarity, respectively. Dashed lines indicate gaps introduced to achieve maximum alignment. The two key FT amino acid residues corresponding to *At*FT Tyr-85 and Gln-140 in *At*FT1 are indicated with black arrows. The three amino acid residues corresponding to Val-70, Ser-76 and Arg-83 in *At*FT, which have been suggested to play an essential role in regulating FT transport, are indicated with white arrows. The five conserved amino acid residues corresponding to Arg-62, The-66, Pro-94, Fhe-101 and Arg-130 in *At*FT, which have been suggested to be required for the 14-3-3 interaction, are indicated with dashed white arrows. The conserved amino acid sequence LGRQTVYAPGWRQN, which distinguishes FT from TFL1 and/or BFT, is overlined in blue.

Sequence comparison indicated that the predicted translated sequence of *MiFT1* from ‘Shelly’ shares 97.2% identity with the translated sequence of *MiFT* from mango cv. Irwin [4], and also corresponds to two similar genes isolated from cv. Alphonso and the Chinese mango cv. SiJiMi [49,50] (see Figure S1A). The translated sequence of *MiFT2* from ‘Shelly’ shared 100% identity with the predicted translated sequence of an EST encoding an *FT-like* gene which we identified in ‘Tommy Atkins’ and ‘Keitt’ mango transcriptomes (see Section 4.2), and 99.4% identity with the translated *MiFT-like* sequence of ‘SiJiMi’, which was named *MiFT3* [50] (see Figure S1B). As shown in Figure 2, ClustalW-based alignment of the translated versions of the *MiFT/TFL1s* identified from ‘Shelly’, together with FT/TFL1 proteins from various other plant species, revealed that *Mi*FT1 contains major characteristics associated with FT florigenic activity [27,33,34]. These include two conserved amino acids (Tyr-89 and Gln-144, corresponding to Tyr-85 and Gln-140 in *At*FT) that have been reported as key residues for promoting flowering, as well as an almost completely conserved FT amino acid sequence—LGRQTVYAP**A**WRQN—in its C-terminal region, where a conservative amino acid substitution, Ala-141, corresponds to Gly-137 in *At*FT. Notably, *Mi*FT1 is also characterized by the existence of three amino acid residues, Val-74, Ser-80 and Arg-87 (corresponding to Val-70, Ser-76 and Arg-83 in *At*FT), which are suggested to have essential roles in regulating FT transport from leaves to the shoot apex [35]. *Mi*FT1 is also characterized by the presence of five additional conserved amino acid residues, Arg-66, The-70, Pro-98, Fhe-105, and Arg-134 (corresponding to Arg-62, The-66, Pro-94, Fhe-101 and Arg-130 in *At*FT), which is suggested to be required for the 14-3-3 interaction [28] (see Figure 2). By comparison, ‘Shelly’ *Mi*FT2 also contains the two important conserved amino acids Tyr-89 and Gln-144, but it is characterized by a semi-conserved amino acid sequence in its C-terminal region—**P**G**K**Q**P**VYAPGWRQN—in which Pro-143, Lys-145 and Pro-147 are present, instead of the expected conserved Leu-128, Arg-130 and Thr-132 (corresponding to *At*FT). Moreover, unlike *Mi*FT1, ‘Shelly’ *Mi*FT2 contains only two conserved amino acid residues, Val-74 and Ser-80, which is suggested to play a role in FT movement. Only three conserved residues, Arg-66, The-70, Pro-98, is suggested to be required for the 14-3-3 interaction (see Figure 2).

With regards to the two *MiTFL1* genes isolated from ‘Shelly’, sequence analysis indicated that their translated sequences share 99.4% identity with the translated versions of two *TFL1* genes isolated from ‘Alphonso’ (*MiTFL1* and *MiTFL1*) [49], and also correspond to the translated sequences of *MiTFL1-1* and *MiTFL1-2* from ‘SiJiMi’ [51] (see Figure S1C,D). A detailed examination of their translated sequences revealed that both clones exhibit 68.5–80.2% similarity to TFL1 proteins from other plant species (not shown), and contain the two crucial conserved amino acid residues, His-85 and Asp-140 (corresponding to *At*TFL1), which are required for their anti-florigenic function [27,33,34] (see Figure 2).

Lastly, the identity of the four isolated genes was also confirmed by phylogenetic analysis, where *Mi*FT1 and *Mi*FT2 proteins were more closely related to other FT-like proteins, whereas *Mi*TFL1-1 and *Mi*TFL1-2 clustered with other TFL1 proteins (see Figure S2). For the benefit of readers from the mango community, we also used the recently published ‘Tommy Atkins’ mango genomic database [52] to search for the corresponding genomic annotations of our four cloned ‘Shelly’ genes, and for the different *MiFT/TFL1* genes previously isolated from distinct mango cultivars [4,49,50,51]. A summary of that search is presented is the supplementary section (see Data S1A–C).

### 2.3. Functional Analysis of Mango cDNA Encoding FT/TFL1 Proteins in Transgenic Arabidopsis Plants

**Figure 3:**
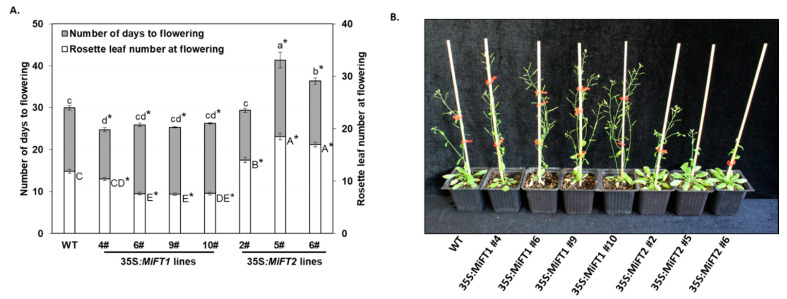
Ectopic expression of ‘Shelly’ *MiFT1* and *MiFT2* genes in *Arabidopsis*. (**A**) Number of days to flowering and rosette leaf’s at bolting of *Arabidopsis* Col-0 (WT), four representative lines constitutively expressing *MiFT1,* and three representative lines constitutively expressing *MiFT2,* under LD conditions. Values represent means ± SE of number of days to flowering and number of rosette leaves at flowering; *n* = 7–34 plants per line. Different small or capital letters indicate significant differences of number of days to flowering or rosette leaf number at bolting, using Tukey–Kramer multiple comparison test, with *p* ≤ 0.05. Asterisks (*) denote a significant difference between the control and a specific 35S:*MiFT1-* or *MiFT2*-overexpressing line, using least significant difference (LSD) test, according to pairwise comparison by Student’s *t*-test, with *p* ≤ 0.05. (**B**) *MiFT1-* and *MiFT2*-overexpressing lines exhibiting early and late flowering phenotypes, respectively, relative to WT plants under LD conditions. Photographs were taken 32 days after sowing.

To further investigate the functions of the isolated ‘Shelly’ *MiFT/MiTFL1* genes in flowering regulation, their cDNA (driven by the cauliflower mosaic virus (CaMV) 35S promoter) was individually ectopically expressed in wild-type (WT) *Arabidopsis* Col-0 plants (see Section 4.3). After transformation with the pART27 35S:*MiFT* constructs, nine independent PCR-positive 35S::*MiFT1* and four independent PCR-positive 35S:*MiFT2* kanamycin-resistant plants were obtained. All of the 35S:*MiFT1* plants were phenotypically distinguishable from the WT plants and exhibited an early flowering phenotype under inductive long-day (LD) conditions. In contrast, the *MiFT2-*overexpressing plants displayed different degrees of late flowering phenotype under the same conditions (not shown). We then used the F_3_ progeny of four 35S:*MiFT1* and three 35S:*MiFT2* homozygous lines for detailed phenotypic analysis, together with the non-transformed control plants. In all 35S:*MiFT1-*transgenic lines, *MiFT1* promoted a significantly early floral transition under inductive LD conditions. In contrast, under the same conditions*, MiFT2* did not induce early flowering (Figure 3). Specifically, the 35S:*MiFT1-*transgenic lines flowered within 25–26 days of seed sowing after producing 7–10 rosette leaves, whereas WT control plants flowered within 30 days of seed sowing, after producing 12 rosette leaves. 35S:*MiFT2* plants flowered within 31–42 days of seed sowing, after producing a significantly higher number of rosette leaves (14–18) than the WT plants. Except for the altered flowering time, no obvious floral phenotypes were observed in either 35S::*MiFT1*- or 35S::*MiFT2-*transgenic plants (Figure 3B).

**Figure 4:**
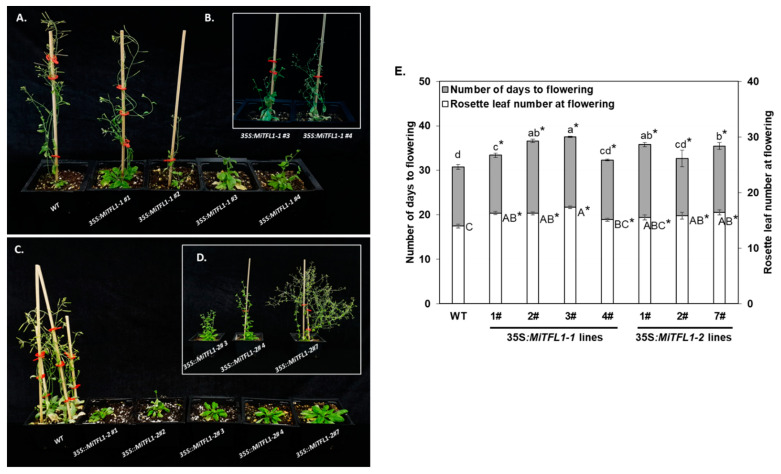
Ectopic expression of ‘Shelly’ *MiTFL1-1* and *MiTFL1-2* genes in *Arabidopsis*. 35S:*MiTFL1-1-* and 35S:*MiTFL1-2*-transformed plants exhibit late flowering phenotype relative to WT plants and display branched phenotype and/or shoot-like inflorescences. Photographs were taken 66 and 72 days from sowing (**A**,**C**), and 94 and 103 days from sowing (**B**,**D**). Comparison of flowering phenotypes of *Arabidopsis* Col-0 (WT), four lines constitutively expressing *MiTFL1-1* and three lines constitutively expressing *MiTFL1-2*, under LD conditions (**E**). Values represent means + SE of number of days to flowering and number of rosette leaves at flowering; *n* = 17–35 plants per line. Different small or capital letters indicate significant differences of number of days to flowering or rosette leaf number at bolting, using Tukey–Kramer multiple comparison test, with *p* ≤ 0.05. Asterisks (*) denote a significant difference between the control and a specific 35S:*MiTFL1-1-* or *MiTFL1-2*-overexpressing line, using least significant difference (LSD) test, according to pairwise comparison by Student’s *t*-test, with *p* ≤ 0.05.

A similar approach was used to investigate the roles of *MiTFL1-1* and *MiTFL1-2*. In this case, four independent PCR-positive 35S:*MiTFL1-1* and seven independent PCR-positive 35S:*MiTFL1-2* kanamycin-resistant plants were obtained, all displaying different degrees of late flowering phenotype under LD conditions. Notably, some of the 35S:*MiTFL1-1*- and 35S:*MiTFL1-2*-transformed plants also exhibited a branched phenotype and/or shoot-like inflorescences (Figure 4B,D). The F_3_ progeny of four 35S:*MiFTFL1-1* and three 35S:*MiTFL1-2* homozygous lines were further used for detailed phenotypic analysis, together with non-transformed control plants. All of the examined 35S:*MiTFL1-1-* and 35S:*MiTFL1-2-*transgenic lines showed a significant delay in flowering transition under LD conditions, reflected in more days required for flowering, along with an increased number of rosette leaves upon flowering compared to the WT control plants (Figure 4E). The degree to which flowering was delayed was more or less similar to that in the *Mi*FT2-overexpressing plants.

### 2.4. Tissue-Specific Expression Patterns of MiFT/TFL1 Genes

**Figure 5:**
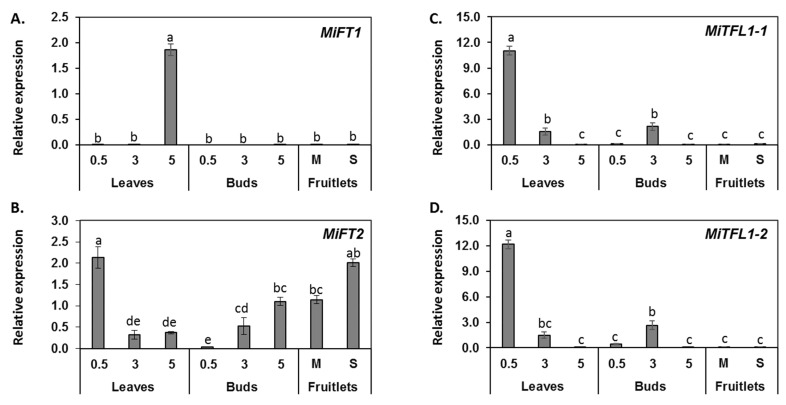
Expression of MiFT/TFL1 genes in various tissues. Expression profiles of *MiFT1* (**A**), *MiFT2* (**B**), *MiTFL1-1* (**C**) and *MiTFL1-2* (**D**) genes in leaves and buds of 0.5- or 3-year-old seedlings and 5-year-old grafted ‘Shelly’ trees, and in fruitlet Mesocarp (M) and Seed (S) tissues. The data are mean of three replicates ± SE. Different letters indicate significant differences according to Tukey–Kramer multiple comparison test, with *p* ≤ 0.05.

To gain further insight into the potential roles played by the isolated *MiFT/TFL1* genes, we examined their specific expression patterns by studying different RNA samples using real-time quantitative PCR (qPCR) with gene-specific primers (see Table S1). We first assessed *MiFT/TFL1-* expression levels in leaf or bud tissues collected in mid-December from trees at different stages of the juvenile-to-adult transition (0.5- or 3-year-old seedlings, and 5-year-old grafted ‘Shelly’ trees). In addition, we measured the expression levels of *MiFT/TFL1* in fruitlet tissues (pericarp and seeds) collected in May from adult trees. As expected, the juvenile (0.5- and 3-year-old) seedlings did not flower, whereas the 5-year-old ‘Shelly’ trees exhibited high flowering rates in the spring (not shown). In line with Nakagawa’s previous observations [4], *MiFT1* was predominantly expressed in the leaves of adult trees. No significant expression of this gene was detected in juvenile leaves, buds of any age or fruitlets (Figure 5A). This pattern fits the suggested role of *MiFT1* as a flowering inducer, produced in leaves of adult trees during the winter.

*MiFT2* was expressed in all examined tissues, but its highest accumulation was in the leaves of the 0.5-year-old seedlings and in the seeds of young fruitlets. As the juvenile seedlings matured, *MiFT2* expression decreased in the leaves and increased in the buds (Figure 5B).

Finally, *MiTFL1-1* and *MiTFL1-2* displayed similar expression patterns. Both transcripts were highly expressed in leaves of 0.5-year-old juvenile seedlings. Both TFL1-encoding genes were also expressed in the buds and leaves of the 3-year-old semi-juvenile trees. By comparison, at the selected sampling point, their expression in the buds and leaves of the adult trees was very low. In addition, both transcripts exhibited very low accumulation levels in fruitlet tissues (Figure 5C,D).

Taken together, while our results confirm previous studies linking *MiFT1* with mango floral induction [4,49,50], they also hint at a possible non-canonical modified function for *MiFT2* that is not related to flower induction. Furthermore, the data on *MiTFL1-1* and *MiTFL1-2* suggest that both genes assume similar redundant roles, repressing transition to flowering, thus corroborating Wang et al.’s data [51].

### 2.5. Monitoring the Expression of MiFT/TFL1 Genes under Different Fruit Load Conditions

**Figure 6:**
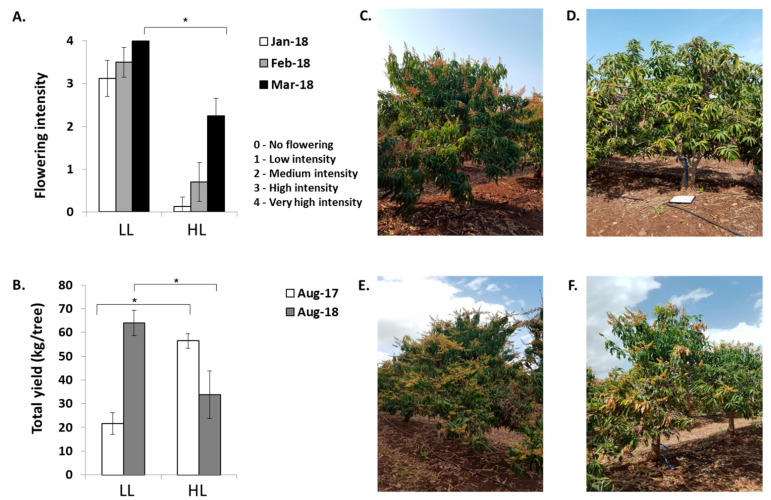
Effect of fruit load on flowering and on the next year’s total yield. (**A**) Flowering-intensity levels. (**B**) Total yield. Uniform HL and LL trees were selected at the beginning of May 2017. Flowering levels were ranked from the end of January 2018 through March 2018, as decribed in Figure 1. Total yield of fruit developing from fruit set in March 2017 was determined in August 2017 and in August 2018 by weighing all fruit harvested from the selected trees. Values represent means ± SE of six trees. Asterisks (*) denote a significant difference at the same time point, or between seasons, using least significant difference (LSD) test, according to pairwise comparison by Student’s t-test, with *p* ≤ 0.05. (**C**–**F**) Images of LL and HL trees selected based on their fruit load intensity at the beginning of May 2017. Photographs were taken in January 2018 (**C**,**D**) and March 2018 (**E**,**F**).

In the spring of 2017, we identified six uniform ‘Shelly’ trees in a commercial orchard with a potentially light load (LL) of fruit, and six uniform trees with a potentially heavy load (HL) of fruit. The fruit were harvested from each tree in August 2017. As shown in Figure 6B, the average yield of the LL trees was 22.3 kg/tree, whereas the average yield of the HL trees was significantly higher (63 kg/tree). From September 2017 to February 2018, we sampled leaves and terminal buds at different times for RNA extraction. The flowering surveys, which were carried out from January through March, revealed that inflorescences appeared earlier in the LL trees than in the HL trees (Figure 6A); the LL trees displayed new developing inflorescences from January to March, while in the HL trees, the vast majority of new emerging inflorescences were mainly detected 2 months later (see Figure 6C,D). Accordingly, the flowering records indicated that in March, the LL trees displayed significantly higher scores than the HL trees (Figure 6A—black columns). Finally, the following year’s yield data (recorded in August 2018) matched the level of the previous spring flowering intensity scores (see Figure 6B—gray columns).

**Figure 7:**
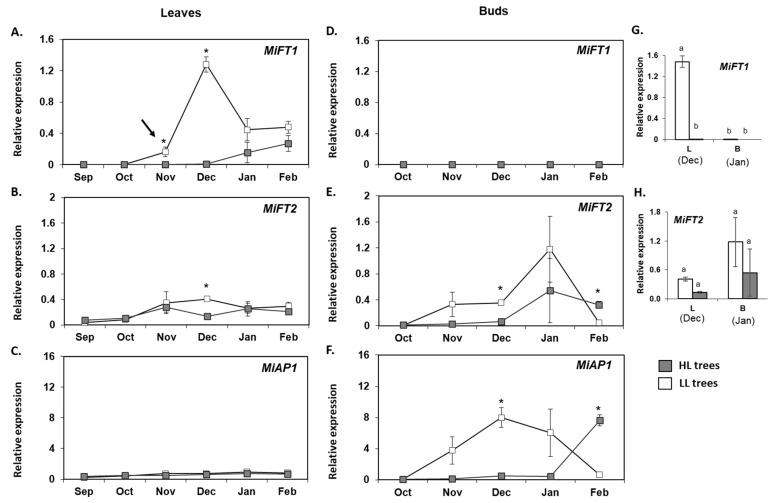
Seasonal expression profiles of *MiFT1*, *MiFT2* and *MiAP1* in leaves (**A**–**C**), and buds (**D**–**F**) of HL (heavy load), and LL (light load) ‘Shelly’ trees. Tissue sampling was performed using six uniform HL and LL trees that were divided into three pairs of trees at the beginning of May 2017. The collected leaves and terminal buds were dissected and pooled to form three biological replicates (for each tissue and tree status). Data are means±SE of three independent replicates. Asterisks (*) denote a significant difference between the transcript expression in HL and LL tissues at the same time point, using least significant difference (LSD) test, according to pairwise comparison by Student’s *t*-test, with *p* ≤ 0.05. Sampling intervals are shown on the *X*-axis. The timing of flower induction is indicated by black arrow. Comparison of *MiFT1* and *MiFT2* expression in leaves (L) vs. buds (B), at those time points at which its expression was maximal (**G**,**H**). Different letters indicate significant differences according to Tukey–Kramer multiple comparison test, with *p* ≤ 0.05.

Next, we carried out real-time qPCR analyses to monitor *MiFT/TFL1* expression patterns in leaves and terminal buds that were sampled from September 2017 to February 2018 from previously LL or HL trees. LL leaves revealed a sharp increase in *MiFT1* expression from November through December, followed by a decrease in transcript accumulation in January and February. In the leaves of HL trees, *MiFT1* followed a different pattern. In this case, *MiFT1* expression was very low until December, and then it marginally increased (Figure 7A). Furthermore, in a parallel examination of *MiFT1* accumulation in the buds, it was nearly undetectable throughout the sampling period under both conditions (Figure 7D). Lastly, a comparison of *MiFT1* transcript accumulation in leaves and buds of HL vs. LL trees, at the time points at which expression was maximal, revealed a significantly higher accumulation in the leaves of the LL trees (Figure 7G).

*MiFT2* expression in leaves increased slightly till November under both fruit load conditions. In December, *MiFT2* expression became significantly higher in the samples of LL trees compared to HL trees (Figure 7B). Expression of *MiFT2* in buds sampled in December was also significantly higher in LL vs. HL trees. In buds, the highest expression for both fruit loads was reached in January. In February, expression in HL buds was significantly higher than in LL buds (Figure 7E). Moreover, a comparison of *MiFT2* expression levels in leaves and buds revealed that unlike *MiFT1*, *MiFT2* accumulation at the time points at which its expression was maximal did not differ significantly between the two tissues, or between trees under different fruit load states (Figure 7H).

Finally, we followed the expression of the downstream *MiAP1* gene, which is expected to accumulate in meristems that are going through floral transition, forming inflorescences [4]. In the leaves, throughout the entire sampling period, *MiAP1* transcript remained stable and low under both fruit load conditions (Figure 7C). In contrast, bud analyses revealed that coinciding with the transient strong upregulation of *MiFT1* observed in the leaves of LL trees from November through December, a strong increment in *MiAP1* transcript was found at those same sampling points in the buds from those trees, followed by a sharp decrease to its initial levels (Figure 7F). On the other hand, concurring with the delayed elevation of *MiFT1* in the leaves under HL conditions, in the buds of those trees, *MiAP1* remained very low until January and only then increased in level.

**Figure 8:**
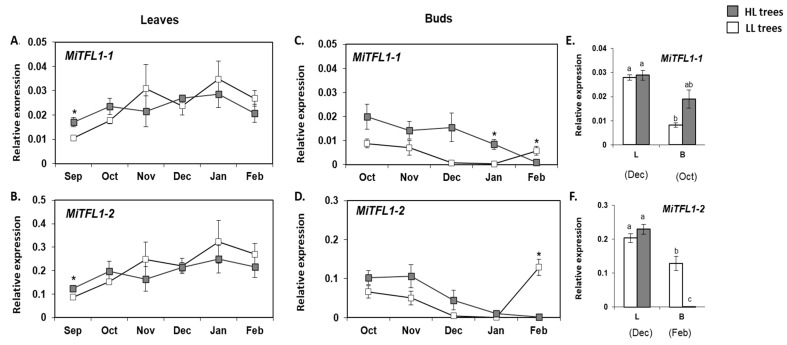
Seasonal expression profiles of *MiTFL1-1* and *MiTFL1-2* in leaves (**A**,**B**), and buds (**C**,**D**), of HL (heavy load), and LL (light load) ‘Shelly’ trees. Tissue sampling was performed using six uniform HL and LL trees that were divided into three pairs of trees at the beginning of May 2017. The collected leaves and terminal buds were dissected and pooled to form three biological replicates (for each tissue and tree status). Data are means±SE of three independent replicates. Asterisks (*) denote a significant difference between the transcript expression in HL and LL tissues at the same time point, using least significant difference (LSD) test, according to pairwise comparison by Student’s *t*-test, with *p* ≤ 0.05. Sampling intervals are shown on the *X*-axis. Comparison of *MiTFL1-1* and *MiTFl1-2* expression in leaves (L) vs. buds (B), at those time points at which its expression was maximal (**E**,**F**). Different letters indicate significant differences according to Tukey–Kramer multiple comparison test, with *p* ≤ 0.05.

*MiTFL1-1* and *MiTFL1-2* expression in leaves from trees under both fruit load conditions displayed a similar moderate upregulation from September to February (Figure 8A,B). Both transcripts only accumulated to slightly higher levels in the HL leaves in September. The bud analyses revealed different results. As shown in Figure 8C,D, starting from October, and coinciding with a decrease in the regional maximum and minimum temperatures (see Figure S3), both *MiTFL1-1* and *MiTFL1-2* exhibited a downregulation pattern, reaching close to null levels in HL trees in February. In LL trees, there was an increase in *MiTFL1-1* and *MiTFL1-2* levels from January to February. Moreover, the rate of decrease in *MiTFL1s* levels was higher in the LL vs. HL trees and therefore in January, *MiTFL1-1* expression remained significantly higher in the HL trees. Finally, a comparison between *MiTFL1-1* and *MiTFL1-2* expression in leaves and buds on the sampling dates with maximal expression showed that overall, both transcripts accumulated to higher levels in the leaves than in the buds (see Figure 8E,F).

## 3. Discussion

The most common version of AB is usually manifested as a repression of flower induction after a heavy-load fruit year, leading to a reduction in the next season’s yield. On the other hand, there are cases in which AB occurs despite a suitable return to flowering. Such is the case, for example, in pistachio (*Pistacia vera* L.), where the alternation between years of high and low fruit load is a consequence of abscission of inflorescence buds on high-yielding trees during the summer [55]. In the case studied here, the results of the de-fruiting experiment confirmed the classical nature of the AB trait in the ‘Shelly’ mango. The results also demonstrated that from May onwards, HFL in ‘Shelly’ attenuated the rate of inflorescence development, but it was only from July onwards that this factor repressed, to some extent, the rate of return to flowering. Previous studies performed in fruit tree species such as citrus [44], olive [56] and avocado [47] have pointed to the existence of a critical physiological window of time, after which fruit load (or the **“**memory**”** of fruit being present on the tree) effectively represses flowering. A noteworthy point is that whereas in avocado and citrus, flowering induction occurs in parallel to the presence of developing fruit on the tree [44,47] In mango, like in olive, flower induction takes place after the fruit is harvested from the tree [4,48]. Consideration of the time interval observed here between presence of the fruit on the trees (during the spring and summer), and the period when flowering induction is expected to occur (in the winter), together with the observed delay in inflorescence appearance with later de-fruiting treatments, suggests that in ‘Shelly’, the “memory” of HFL on the tree is preserved after fruit removal. This both delays and partially suppresses flowering.

HFL or the “memory” of HFL has been shown to affect *FT/TFL1-like* gene expression in leaves, buds or both in different fruit trees [4,40,44,46,47,48]. Here, we cloned two *FT-like* and two *TFL1-like* genes from mango ‘Shelly’ cultivar. Cloning of the ‘Shelly’ *MiFT/TFL1* genes allowed us to further investigate their expression patterns at various stages of the tree’s life cycle, as well as their spatial and temporal expression profiles, i.e., prior to and during inflorescence development, in both leaves and terminal buds of trees under different fruit load conditions. We also studied the effect of overexpressing each of the cloned genes in *Arabidopsis*.

One of the FT-encoding genes in ‘Shelly’—*MiFT1—*is annotated in the ‘Tommy Atkins’ mango genomic database [52] as Manin02g003570.1, located on chromosome 2 and corresponding to the previously described *MiFT* from ‘Irwin’ [4], *MiFT1* from ‘Alphonso’ [49], and *MiFT1* from ‘SiJiMi’ [50] (Data S1A,B). The encoded protein appears to contain all known characteristics associated with FT flowering-induction activity [27,33,34]. *MiFT1* was mainly expressed in leaves of adult ‘Shelly’ trees, and its ectopic expression in *Arabidopsis* resulted in early flowering. Our expression and transgenic results thus corroborate earlier reports suggesting that *MiFT1* acts as the major factor controlling flower induction [4,49,50]. Furthermore, similar to Nakagawa et al. [4], our analysis showed that starting in November, accumulation of *MiFT1* in LL trees transiently increased in the leaves, and was accompanied by a parallel upregulation of the floral marker gene *MiAP1* in the buds. On the other hand, concomitant with a reduction in flowering levels, *MiFT1* upregulation was delayed and reduced in the leaves of HFL ‘Shelly’ trees, resulting in parallel delayed and reduced upregulation of *MiAP1* in the buds.

The second FT-encoding gene cloned from ‘Shelly’, which we termed *MiFT2*, is annotated in the mango genome database as Manin03g001830.1, located on chromosome 3; it corresponds to the previously described *MiFT3* from ‘SiJiMi’ [50] (Data S1A,B). FT paralogs that sometimes exhibit different expression patterns or functions are well documented. For instance, in apple, two *FT* paralogs appear to share redundant floral-promoting roles; whereas *MdFT1* is expressed in the apical meristem during the floral transition, *MdFT2* peaks at a later stage in the reproductive organs [57]. On the other hand, in Satsuma mandarin, whereas *CiFT3* is expressed mainly in the stems and leaves and is suggested to act as the major factor controlling flowering, two other *FT* homologs (*CiFT1* and *CiFT2*), which are highly expressed in young fruitlet tissues, are not considered to be involved in flowering induction [58]. Our results, based on sequence analysis, expression patterns and phenotype resulting from overexpression in *Arabidopsis* suggest that *Mi*FT2 acts as a flowering repressor. We note that this conclusion is not in full agreement with the results presented for *MiFT3* from ‘SiJiMi’ (encoding the corresponding protein), where evidence suggests that it encodes a flowering inducer, albeit a weaker one than *MiFT1* [51]. However, our data are most consistent with the notion that in ‘Shelly’, *Mi*FT2′s role is not related to flower induction. In particular, its putative protein comprises a semi-conserved B segment, characterizing different *FT* homologs that have acquired flowering-repression functions during evolution [18]. Furthermore, whereas *MiFT1* was mainly expressed in the leaves of adult trees, *MiFT2* was very strongly expressed in the leaves of young seedlings in their juvenile phase, as well as in fruitlet tissues and adult tree buds. *MiFT2* was also similarly expressed in the leaves and buds of trees under different levels of fruit load. Lastly, ectopic expression of *MiFT2* delayed flowering in *Arabidopsis*.

With respect to the TFL1-encoding genes, the ‘Shelly’ TFL1 gene *MiTFL1-1* is annotated in the mango genome database as Manin19g005780.1, located on chromosome 19, and corresponds to the previously described *MiTFL1α* from ‘Alphonso’ [49] and *MdTFL1-1* from ‘SiJiMi’ [51]. The ‘Shelly’ TFL1-encoding gene termed here as *MiTFL1-2* corresponds to the previously described *MiTFL1* from ‘Alphonso’ [49] and *MdTFL1-2* from ‘SiJiMi’ [51]. This gene is not annotated in the ‘Tommy Atkins’ mango genomic database [52], however, we were able to locate its sequence on chromosome 3 (see Data S1A,C). The putative proteins encoded by both genes display the crucial conserved amino acid residues required for TFL1 activity [49,51]. Moreover, coinciding Wang et al. [51] who showed that overexpression of *MiTFL1*s from ‘SiJiMi’ delayed flowering in *Arabidopsis*, here we also confirmed that both *MiTFL1* genes from ‘Shelly’ inhibit flowering when ectopically expressed in *Arabidopsis*. In addition, we noticed that in some overexpressing lines, *MiTFL1-1* and *MiTFL1-2* induced a highly branched phenotype, affecting plant architecture. This branching phenotype was not reported upon overexpression of *MiTFL1s* from ‘SiJiMi’ in *Arabidopsis*, but is well described in the literature [21,31].

As already noted, *TFL1* genes act to repress floral transition in different types of meristems, regulating both plant juvenility and flowering time [30,31]. For instance, in citrus, increased levels of *CsTFL1* were shown to be well-correlated with juvenility, because its transcript accumulated to higher levels in juvenile stem tissues than in adult tissues [59]. *PoTFL1-like* genes were also recently shown to be highly expressed during the juvenile phase in passion fruit (*Passiflora* spp.) [60]. Similarly, our expression analysis revealed that both *MiTFL1* transcripts were preferentially expressed in leaves and apical meristems of ‘Shelly’ seedlings, linking them with the control of mango juvenility.

Moreover, current studies in fruit trees, including Japanese apricot and loquat, have demonstrated that *TFL1* genes can be detected not only in juvenile tissues, but also in leaf buds or in tree leaves at their mature stage [61,62]. In line with these reports, it was recently shown that four *MiTFL1* genes, showing similar expression patterns, are expressed in both vegetative and reproductive tissues of adult ‘SiJiMi’ mango trees [51]. Here, our expression analysis showed fluctuations in the expression patterns of the two cloned *MiTFL1* genes in the leaves and buds of adult trees. From September to January, accumulation of *MiTFL1*-*1* and *MiTFL1-2* transcripts increased similarly in leaves under both fruit load conditions. On the other hand, a downregulation pattern of the two *MiTFL1* transcripts, which was associated with a decrease in regional temperatures, was observed in the buds of trees under both conditions. Despite these similarities, *MiTFL1s* downregulation was delayed, to some extent, under HFL conditions, suggesting that *de novo MiTFL1*s expression in the buds affects flowering transition.

Taken together, our results reinforce the notion that in mango, similar to other evergreen fruit tree species, a putative low temperature-based signal results in the upregulation of *FT-like* genes, which have floral-promoting functions [4,40,44,47]. In addition, they imply that this putative low temperature signal might also induce downregulation of *MiTFL1* genes. The requirement of a prolonged cold period to flower is perhaps best documented in *Arabidopsis*, where cold temperature (vernalization) triggers flowering through epigenetic silencing of the floral repressor gene *FLOWERING LOCUS* C (*FLC*) [63]. Interestingly, recent studies in citrus have identified a gene encoding a protein similar to FLC (*CcMADS19*), with increased expression under HFL, together with epigenetic changes [64]. Those authors suggested that the increase in this gene’s expression is regulated by epigenetic changes similar to FLC. They also suggested that *Cc*MADS19 accumulation under HFL might repress flowering by inhibiting the increase in FT-encoding gene expression during the winter [64]. Whether a similar mechanism operates in mango remains to be established. Furthermore, experimental data have been reported from other species indicating that downregulation of *TFL1* genes associated with a decrease in ambient temperature, reduced fruit load, or both correlates with flowering. For example, studies in woodland strawberry (*Fragaria vesca*) have shown that a reduction in ambient temperature (<13 °C) is required for downregulation of *FvTFL1* in the shoot apex, so that flowering can occur independently of photoperiod [65]. Similarly, in an accession of *F. vesca* endemic to northern Norway, very low temperatures were shown to be required to downregulate *FvTFL1* prior to flowering [66]. Moreover, studies in citrus and olive have demonstrated that HFL prevents downregulation of TFL1-encoding genes in the buds [40,45]. At the same time, different flowering responses to various fruit loads, correlating with distinct degrees of *TFL1*-encoding gene accumulation, were detected in different apple cultivars [48]. Bearing these observations in mind, our results suggest that *MiTFL1* downregulation is induced by low temperature, but that HFL delays this response. Side by side, in view of the opposite *MiTFL1* and *MiAP1* expression patterns observed in the buds (Figure 8C,D vs. Figure 7F), and knowing that in *Arabidopsis*, AP1 suppresses *TFL1* expression in emerging floral meristems [67], an alternative or additional scenario might involve the possibility that *Mi*AP1 accumulation in mango buds impacts their *MiTFL1* expression.

Lastly, as already noted, studies have suggested that the regulation of GA metabolism by fruit load affects mango flowering induction [4]. Additional mango studies have shown that paclobutrazol treatments, which decrease GA content, also lead to an increase in sugar and abscisic acid (ABA) levels in leaves and buds [14,68,69]. Interestingly, accumulation of ABA in the leaves has been recently suggested to impact flowering in mandarin trees by affecting FT-encoding gene expression [70], whereas studies in apple have shown that GA treatment, which affects flowering, results in upregulation of TFL1-encoding genes in the buds [48]. The possibility that fruit load confers changes in the GA:ABA ratio, or in carbohydrate levels, directly or indirectly impacting *FT/TFL1* gene expression in ‘Shelly’, thus warrants further exploration.

## 4. Materials and Methods

### 4.1. Plant Material and De-Fruiting Treatments

Fifteen-year-old ‘Shelly’ mango trees, grafted on ‘13-1’ rootstocks and grown in a commercial orchard at Almagor in the northern district area of Israel (32°50′ N 35°36′ E), were used for the experiment. The fruit load intensity of trees in the orchard was determined at the beginning of May 2017. Sixteen uniform heavily producing trees were randomly assigned to different de-fruiting treatments in groups of four trees (replicates) per treatment (different dates of fruit removal). Complete de-fruiting treatments were performed manually at the beginning of May, June and July 2017. In the fourth treatment, fruit were collected during the commercial harvest season (August 2017). The intensity of flowering was evaluated upon the appearance of the first inflorescences, starting at the end of January 2019 to March 2019, using a blind test in which two surveyors independently scored each tree from 0 (no flowering) to 4 (very high flowering intensity). Lastly, the total yield of fruit that developed from the inflorescences of March 2017 was determined in all treated trees in August 2018, by weighing all of the fruit harvested from individual trees.

### 4.2. Cloning of MiFT/MiTFLs from Mango ‘Shelly’ Cultivar

A previously published annotated sequence of *MiFT* from mango cv. Irwin (AB671587.1), and two annotated sequences of *MiTFL1*s from mango cv. Alphonso (KF258590 and KU206290), were utilized in this study. We also made use of an additional sequence annotation, putatively encoding for an *FT-like* gene (mango_rep_c5502), which was identified in a search conducted against ‘Tommy Atkins’ and ‘Keitt’ mango transcriptomes [71]. The four corresponding ‘Shelly’ cDNAs were isolated by real-time qPCR using pairs of end-to-end primers, designed within the 5′ and 3′ UTRs of each sequence annotation (see Table S1), and cDNA synthesized from ‘Shelly’ tissues as the template (a mixture of samples collected from leaves and buds at different time points). The obtained PCR products were ligated into the CloneJET vector (Fermentas, Vilnius, Lithuania), sequenced (Hy-labs Laboratories, Rehovot, Israel), and further used as templates to generate constructs for *Arabidopsis* transformation purposes.

### 4.3. Arabidopsis Transformation and Phenotypic Analysis

For constitutive expression of the *MiFT/TFL1* genes in *Arabidopsis*, the plasmid pART7-based pART27 vector was used. The protein*-*encoding regions of the *MiFT/TFL1* genes were first amplified using *MiIFT1/MiIFT2*-*EcoR*I and *MiIFT1/MiIFT2*-*Xba*I, or *MiITFL1-1/MiITFL1-2*-*EcoR*I and *MiITFL1-1/MiITFL1-2*-*BamH*I primers, respectively (Table S1). The purified PCR fragments were next digested with *EcoR*I and *Xba*I, or with *EcoR*I and *BamH*I, respectively, and cloned into the corresponding sites of the pART7 vector, between the 35S*CaMV* promoter and the *ocs* 3′ transcription terminator. The expression cassettes were then *Not*I-excised from the pART7 constructs and inserted into the binary plant transformation vector pART27. The resulting plasmids were further used for stable transformation of WT (Col-0) *Arabidopsis* plants using the *Agrobacterium tumefaciens*-mediated floral dip method [72]. The transformed seeds were selected on medium containing half-strength Murashige and Skoog salts and kanamycin (50 μg/mL). After transforming with pART27, the third generation of three or four randomly selected transgenic lines for each gene was used for phenotypic assessment of flowering times, along with WT plants which served as controls. Plants were placed in a growth room under a LD regime (25 °C, 16/8 light/dark). Flowering times were measured by counting the number of rosette leaves and the number of days from sowing to bolting.

### 4.4. Plant Material and Tissue Collection from Juvenile and Adult Trees

To compare the expression patterns of *MiFT/TFL1* genes in adult vs. juvenile tissues, we used three 5-year-old ‘Shelly’ mango trees (adult ‘Shelly’ trees grafted on ‘13-1′ rootstocks), and fifteen 0.5-year-old and nine 3-year-old potted ‘Shelly’ seedlings (juvenile trees, germinated from seeds). The trees were grown in an experimental orchard and in a net-house (adult and juvenile trees, respectively) at the Volcani campus, Bet Dagan, Israel (32°6N 34°49E). During the floral induction period, in December 2019, tissue sampling (leaves and terminal buds from adult trees, and leaves and apical meristems from juvenile trees) was carried out early in the morning. Samples of the adult trees (three biological replicates) were immediately frozen in liquid nitrogen, brought to the laboratory and kept at −80 °C until further analysis. For juvenile trees, tissue samples were first pooled to form three biological replicates (each comprising leaves or apical meristems, collected from five 0.5-year-old or 3-year-old seedlings, respectively). Lastly, during May 2020, fruitlet tissues (pericarp and seeds), were collected from the adult trees, frozen in liquid nitrogen and stored at −80 °C.

### 4.5. Tissue Collection from Fruit Trees with Heavy and Low Fruit Loads

Fifteen-years-old ‘Shelly’ mango trees, which were grafted on ‘13-1’ rootstocks and grown in a commercial orchard at Almagor, were used to investigate the effect of fruit load on the expression patterns of *MiFT/TFL1* genes and on flowering. Six uniform HL trees and six uniform LL trees were selected at the beginning of May 2017 for tissue sampling. The total yield of the selected trees was first determined in the commercial harvest season, in August 2017, by weighing all of the fruit harvested from individual selected trees. Following harvest, as part of the agronomical practice carried out in the commercial orchard, all trees were pruned and new emerging shoots were tagged on each tree in September 2017. For sampling purposes, the HL and LL trees were divided into three pairs of trees (three biological replicates). HL and LL tree tissue sampling was carried out at various intervals (from September 2017 to February 2018) using the tagged shoots. Collected leaves and terminal buds of the sampled shoots were dissected in the orchard, pooled to form three biological replicates (for each tissue and tree status), frozen in liquid nitrogen, transported to the laboratory and stored at −80 °C for further analysis. To maintain a uniform sampling pattern, dissection of three to four leaves was performed from the fourth leaf relative to a reference point, set as the base of a newly born shoot. The intensity of the flowering in the selected trees was then evaluated at various time intervals, starting from the end of January 2018 until March 2018, as described in Section 4.1. The total yield of individual trees carrying fruit that developed from the flowers of March 2018 was determined in August 2018.

### 4.6. RNA Isolation and cDNA Synthesis

Total RNA from leaves and terminal buds was extracted using a Plant/Fungi Total RNA Isolation Kit (NorgenBiotekcrop, Thorold, ON, Canada) following the manufacturer’s instructions. RNA was quantified using a NanoDrop ND-100 spectrophotometer (NanoDrop Technologies, Rockland, ON, Canada). Total RNA (4 μg) pretreated with 1 unit of RQ1 DNase served as the template in the synthesis of first-strand cDNA, using an anchored oligo-dT primer and Verso cDNA Synthesis Kit (Thermo Scientific, Waltham, MA, USA) according to the manufacturer’s instructions. The reaction products were used for further analyses.

### 4.7. Real-Time qPCR Analysis

The accumulation of *MiFT1*, *MiFT2*, *MiTFL1-1*, *MiTFL1-2* and *MiAP1* genes in the sampled tissues was evaluated by real-time qPCR using Fast SYBR Green Master Mix (Applied Biosystems, Foster City, CA, USA). Reactions were carried out using 3 μL of the cDNA products (1:10 dilution), 6 μL of SYBR Green PCR Master mix, and 200 nM primers from the relevant specific primer pair (see Table S1), in a final volume of 12 μL. Analysis was performed with a StepOnePlus Real-Time PCR system (Applied Biosystems). A dilution series of CloneJET plasmids, containing the full length of the relevant amplified fragment, was created and standard curves for each gene were established using the pairs of specific primers. The cDNA samples were analyzed in triplicate, with each reaction being subjected to melting-point analysis to confirm the presence of single amplified products. Transcript levels in each sample were estimated using a standard curve for each gene and normalized against the level *MiEF* transcript. The R^2^ values and PCR efficiency obtained for the standard curves of the examined genes are presented in Table S2. Relative expression levels were calculated by dividing each individual gene copy number by the *MiEF* copy number.

### 4.8. Statistical Analysis

Data from different sets of experiments were analyzed using JMP software (JMP Pro14, SAS Institute, Cary, NC, USA). Statistical analyses of the phenotypic data of the 35S:*MiFT/TFL1* lines and *Arabidopsis* WT plants, mango flowering records, and real-time qPCR data of *MiFT/TFL1* genes in various mango tissues, were performed using one-way analysis of variance (ANOVA) by the Tukey–Kramer multiple comparison test, with *p* ≤ 0.05. Additional analyses were performed by the least significant difference (LSD) test, according to pairwise comparison by Student’s *t*-test, with *p* ≤ 0.05.

## 5. Conclusions

Based on our results, we propose that in the mango ‘Shelly’ cultivar, a decrease in ambient temperature induces both an increase in *MiFT1* levels in the leaves, and a decrease in *MiTFL1* accumulation in the buds. On the other hand, HFL also affects *MiFT/MiTFL1* gene expression by suppressing *MiFT1* upregulation and delaying *MiTFL1* downregulation. As a result, flowering is repressed, but not abolished. *Mi*FT2 might operate together with the *Mi*TFL1s to regulate juvenility, whereas in the adult phase, *Mi*FT2 activity might not be related to flowering induction. Further elucidation of the mechanisms by which changes in ambient temperature, together with fruit load, affect the regulation of flowering could encourage the development of trustable agricultural practices that will reduce AB while enhancing ‘Shelly’ cultivar profitability. In particular, with the expected warmer winters, growers will benefit from an improved understanding of these mechanisms.

## Data Availability

Not applicable.

## Supplementary Materials

The following supporting information can be downloaded at: https://www.mdpi.com/article/10.3390/plants11182409/s1, Figure S1: Structural features of ‘Shelly’ *Mi*FT1, *Mi*FT2, *Mi*TFL1-1 and *Mi*TFL1-2 and amino acid sequence comparison with the corresponding FT/TFL1-like proteins from distinct mango cultivars. Figure S2: Phylogeny of *Mi*FT/TFL1s and FT, TSF, MFT, TFL and BFT proteins from various plant species. Figure S3: Minimal and maximal temperatures recorded from September to February at Kfar Nahum near Almagor, where the experimental ‘Shelly’ trees were grown. Data S1: ‘Shelly’ *MiFT/TFL1*cDNA sequences, genomic annotations and their corresponding genes from different mango cultivars. Table S1: Primers used for *MiFT/MiTFL1* gene isolation, real-time qPCR analysis and plasmid preparation. Table S2: R2 values and PCR efficiency obtained for the standard curves of the examined genes.
