# Supplementary material for: Expression Profiling of Four Mango FT/TFL1-Encoding Genes under Different Fruit Load Conditions, and Their Involvement in Flowering Regulation
Source: Plants (Basel). 2022 Sep 15;11(18):2409. doi: 10.3390/plants11182409 (PMC9506463; doi:10.3390/plants11182409)
Supplement: Supplementary file 1 [file plants-11-02409-s001.zip › Gafni et al. Data S1 FINAL.pdf]

Data S1.

A. 'Shelly' *MiFT/TFL1* cDNA sequences

The four cloned 'Shelly' *MiFT/TFL1* cDNAs and their putative protein sequences are presented. For each cDNA sequence, the ATG codon and stop codon are in red font. Forward and reverse primers used for full-length cDNA cloning are highlighted in blue. Forward and reverse primers for real-time qPCR are highlighted in gray.

MiFT1-'Shelly' CDS sequence

TTTACT**ATG**TCTTGGGAAGGCTGTTGTTCCGATAGAGATCCTCTTGTTGTTGGGAGAGTAATTGGAGATGTTATTGACAATTTTAATACGTCAATTTCTCGTAATGT
TAGCTATGGCAACAGGGATGTCGGCAATGGTGTTGAGCTCAAGCCCTCTGTTGTTGCCAATCATCCGAGGGTTGACATTGGTGGAACTGATCTTAGGACCTTCTATA
CTTTGGTCATGGTGGATCCTGATGCACCTAGCCCTAGTAATCCAGGTCTCAGAGAATACTTGCATTGGTTGGTGACTGATATTCCCGGATCTACAGGGGCATCCTTC
GGACAAGAGATTGTGAATTATGAGAGCCCGAGACCAACATTGGGGATTCACAGGTTTGTATTTATGTTGTTTCGTCAACTGGGAAGACAAACTGTGTATGCACCAGC
GTGGCGCCAGAATTTCATCACCAGAGACTTTGCTGAGCTTTACAATCTGGGATCTCCTGCGGCTGCTGTTTACTTCAATTGCCAGAAGGAGACTGGCTCTGGAGGAA
GAAGAAGGCAG**TAA**TATGCTTATGT

MiFT1-'Shelly' protein sequence

MSWEGCCSDRDPLVVGRVIGDVIDNFNTSISRNVSYGNRDVGNGVELKPSVVANHPRVDIGGTDLRTFYTLVMVDPDAPSPSNPGLREYLHWLVTDIPGSTGASFGQ

EIVNYESPRPTLGIHRFVFMLFRQLGRQTVYAPAWRQNFITRDFAELYNLGSPAAAVYFNCQKETGSGGRRRQ

MiFT2-'Shelly' CDS sequence

CATC**ATG**GATGGAGAGGACTATGGTGAAAGTGCTTCCAGGGAGACTAGGGAAAGTGCTTCCAGAGATCCTCTTGTTGTTGGGCGAGTTGTGGGAGACGTTCTTGACA
ACTTTACTAAGTCTATTGCTTTCACTGCTAGCTATGGCCACAAGCTGGAAGTTCACAATGGTGTTTTACTCAAGCCCTCTCAAGTTGCCAAACAACCTAGGGTTTCT
ATTGGTGGAAGTGATTTCAGGAACTTCTACACTTTGGTCATGGTGGATCCTGATGCACCCAGTCCAAGTGACCCCCACCTTAAAGAGTACTTGCATTGGTTGGTGAT
TGATATTCCAGAAAGTACAGGAGCAACTTATGGCAAAGAGGTTGTTTCTTATGAGAGCCCAAAGCCAACAGTGGGGATTCACAGGTTTGTTTTTGTGCTGTTTCGGC
AACCGGGGAAACAGCCTGTGTATGCACCAGGGTGGCGCCAGAACTTCGTAACCAGAGACTTTGCTGAGCTTTACAATCTTGGCTCCCCCGTGGCTGCCCTTTTTTTC
AATTGCCAAAAGGAGACTAAGACCTCTACTAGCTCATCATCAAGCTCAAGGAGACGC**TAG**CTAATTAATATATACATATAACTCCACATATATGGCTCGATGCATGC
ACTTTAACTTCATGATCTCTCCGTGATTGCCTAACAATAATTATAGGCTGTCATGTAGAGACTTC

MiFT2-'Shelly' protein sequence

MDGEDYGESASRETRESASRDPLVVGRVVGDVLDNFTKSIAFTASYGHKLEVHNGVLLKPSQVAKQPRVSIGGSDFRNFYTLVMVDPDAPSPSDPHLKEYLHWLVID
IPESTGATYGKEVVSYESPKPTVGIHRFVFVLFRQPGKQPVYAPGWRQNFVTRDFAELYNLGSPVAALFFNCQKETKTSTSSSSSSRRR*

MiTFL1-1 -'Shelly' CDS sequence

CTCCTACTGATTCA**ATG**GCAAGAATAGTAGACCCTCTTGTTGTGGGGAGAGTGATAGGAGATGTTCTTGATTCTTTCTCTCCAACAATCACAATGTTTGTAAGTTAC
AATAACAGGCAAGTCTGTAATGGCCATGAGCTTTTACCTTCCACAGTTTCCTTCAGACCTAGGGTTGAGATTCAAGGAGGTGATATGAGAACTTTCTTTACACTGGT
GATGACAGACCCAGATGTTCCTGGACCTAGTGATCCTTACTTAAGGGAGCACCTGCACTGGCTAGTGGCAAACATACCAGGAACAACAGATGTCACATTTGGGAGGG
AACTGGTGAGTTATGAGATACCAAGGCCAAATATAGGCATCCACAGATTTGTTTTTGCTCTATTCAGGCAGAAAGGTAGACAGATATTTAACCCACCTTCTTCAAGG
GATAACTTCAGCACTCGAATTTTCGCTGCTGAAAACGATCTCGGTCCTCCTGTTGCTGCCGTCTACTTCAACGCACAAAGAGAAACTGCAGCTAGAAGACGC**TAA**CC
AGATCCCCACATCCCCCATTTTCGTCCAGTCTATTTGGAAACC

MiTFL1-1 'Shelly' protein sequence

MARIVDPLVVGRVIGDVLDSFSPTITMFVSYNNRQVCNGHELLPSTVSFRPRVEIQGGDMRTFFTLVMTDPDVPGPSDPYLREHLHWLVANIPGTTDVTFGRELVSY
EIPRPNIGIHRFVFALFRQKGRQIFNPPSSRDNFSTRIFAAENDLGPPVAAVYFNAQRETAARRR*

MiTFL1-2 'Shelly' CDS sequence

TTCCATGGATCTAACTACCTTCTCTACACTATAAATAGGGAGCTTCAACTTCAAGGGAAGATAAGTAAAAGAAAGAAAAACACATAATTAGATCATTCTCTCTCTCC
A**ATG**GCAAGAATGCCATCAGATCCTCTTGTAGTTGGCAGAGTGATTGGAGATGTTGTTGATTGTTGTGATCAAACTGTCAAAATGGCAGTCACCTACAACTCTTCCA
AGCAGGTGTACAATGGCCATGAGCTGTTTCCATCTTCAGTGACTGTGAAACCTAAGGTTGAGGTTCACGGAGGTGATATGAGATCATTCTTCACATTGATCATGACA
GACCCAGATGTTCCTGGTCCAAGTGACCCATACTTGAGGGAGCATTTACACTGGGTGGTGACAGACATCCCAGGCACAACTGATGCCACGTTTGGAAGGGAATTGGT
GAACTATGAAATGCCGAGGCCAAATATTGGGATTCACAGGTTTGTTTTCCTTCTGTTCAGGCAGAAACGCAGGCAAACAGTGATAAGTATACCATCTTCAAGGGACG
GCTTCAACACAAGAAAGTTTGCTGAAGACAATGACCTTGGTCAGCCTGTTGCAGCTGTGTTCTTCAATGCACAAAGGGAAACAGCCGCAAGAAGGCGC**TGA**TAAATC
AGGAAATAATTATAAAAAATAAGGTACTATTCCAGAAGAAAAAAATATTTAAAACATTTTAATTTGAAAAGGCGATGGGTGCAG

MiTFL1-2 'Shelly' protein sequence

MARMPSDPLVVGRVIGDVVDCCDQTVKMAVTYNSSKQVYNGHELFPSSVTVKPKVEVHGGDMRSFFTLIMTDPDVPGPSDPYLREHLHWVVTDIPGTTDATFGRELV
NYEMPRPNIGIHRFVFLLFRQKRRQTVISIPSSRDGFNTRKFAEDNDLGQPVAAVFFNAQRETAARRR*

B. *MiFT* genomic annotations and their corresponding genes from different mango cultivars.

MiFT annotations from the mango genomic database (https://mangobase.org/easy_gdb/index.php), and their corresponding genes from 'Irwin' (1), 'Alphonso' (2), 'SiJiMi' (3) and 'Shelly' (this study).

The predicted exons are indicated in blue font, based on the updated annotations in the mango genomic database. Manin03g001820.1 and Manin03g001830.1 annotations require further validation.

	***MiFT-like* genes' genomic annotations**	**'Irwin' cloned *FT* genes (1)**	**'Alphonso' cloned *FT* genes (2)**	**'SiJiMi' cloned *FT* genes (3)**	**'Shelly' cloned *FT* genes (this study)**
1	Manin02g003570.1		*MiFT1* (JX177434.1)		
2	Not found	*MiFT* (AB671587.1)	*MiFT3*[* #]	*MiFT1* (MT419778)	*MiFT1* (OP341600)
3	Manin03g001820.1		*MiFT2* (JX141437.2)	*MiFT2* (MT419779)	
4	Manin03g001830.1			*MiFT3* (JQ700254.1)	*MiFT2* (OP341601)

[#] Accession numbers not assigned.
*The sequences of *MiFT1* and *MiFT3* from 'Alphonso' are identical, but have different 5' UTRs.

(1). Nakagawa, M.; Honsho, C.; Kanzaki, S.; Shimizu, K.; Utsunomiya, N. Isolation and Expression analysis of FLOWERING LOCUS T-like and gibberellin metabolism genes in biennial-bearing mango trees. Sci. Hortic. 2012, 139, 108–117. doi:10.1016/j.scienta.2012.03.005.
(2). Vyavahare, S.N.; Krishna, B.; Joshi, S.S.; Chaudhari, R.S.; Subramaniam, V.R.; Sane, P.V. Characterization of mango Flowering Locus T (FT) and Terminal Flower 1 (TFL1) genes. Acta Hortic. 2017, 1, 113–124. doi:10.17660/ActaHortic.2017.1183.16.
(3). Fan, Z.-Y.; He, X.-H.; Fan, Y.; Yu, H.-X.; Wang, Y.-H.; Xie, X.-J.; Liu, Y.; Mo, X.; Wang, J.-Y.; Luo, C. Isolation and functional characterization of three MiFTs genes from mango. Plant Physiol. Biochem. 2020, 155, 169–176. doi:10.1016/j.plaphy.2020.07.009.

> Manin02g003570.1

ATGTCTTGGGAAGGCAGTTGTTCCAATAGAGATCCTCTTGTTGTTGGGAGAGTAATTGGAGATGTTATTAACAATTTTAATAGGTCAGTTTCTCTTAATGTTAGCTA
TGGCAACAGGGATGTCAACAATGGTGTTGAGCTCAAGCCTGCTGTTGTTGCCAATCATCCAAGGGTTGACATTGGTGGAACTGATCTTAGGACCTTCTATACTTTGG
TATGCCTCAACATTCTTTTTTGTTACCTCTTATTAAACATAATAATTATGTGTATATTTACATCCATGTTTTTATGAATCTACGTAGTAATGTATATACGAGATAGC
ATTATTTCTCACAGATATACTAATTGTATATACAAGAACCTGTTTTGCATGTTCGTTTTTTTTTTTATTATTTTCTAAAAACTACTTTATGTATTTGCATCATCCNN
NNNNNNNNAAAAAAAAAAAAACTACTTCTGAATGAACTTACAACATCTTGTTTGGCAGATGTTCAAGTTAAAAAATCCAGTTCTTAAAGTATATACAAAATCCTATTT
TACAACTGAATGCACTGATATATATTTTTTTCTAATACAATAACATATAGAAATTATGAAATTTTATCTTCTGAGGAAAGACAAACTTTCACGTTTATGATTCTTTC
TCAGGAGAGTTGATTCATGCATGAATATTAGATTTATAATTGATTATTTATGTATTAATCATAAAAATAAAACATGCAGGTCATGGTGGATCCTGATGCACCTAGCC
CTAGTAATCCAAGTCTCAGAGAATACTTGCATTGGTAAGTAGCTTCTGTCTCTCATAATCAACTTCTTTTCTTCTTTTGTTTACACAATGGATAATAAGTTAAACTA
AATTATCGTATCTTTTTACGTGAATAACTACCTTCTGTTTTGCGTTGGCTGAAACTTTCTCTATATCATGGCAAAGATTGAAGGAAGAAGAACCACCGAGAAATAAA
AGCAACTTTGTTGTTCTTGGTTTCTGACATTATTTTATAAAACTACAGCATAAATTTGACAAGTTTTTTAGGTTTATCTCAATAGAATTATTGGAGCACTTTATCAA
CACAGAACAAGGAACATAGGAAAAAAGTGAAACCCAAAATTTCCCCATTTGATATCAAGGAACAACTATTAGATTGGACTGAATAACAACTAGGGAATTGTATCATG
TTCAGAGATTGACTAGTCTAAACTTCATTAAAAAACTGATAAAGCCTGTACAATGATAACTTGTGAAACAAAGAAAAAAGGGCGGTTTCTCACAAACGTTTGTTTCT
AACATTATCTTTTTGTGCTTAATTTTCTCTTTGGGATAATACTATGAATTTTGTAATGCCTACATATTGTTTAATTGTTCTTGAAATTGATCTGAACTTGTTAGGTT
GGTGACTGATATTCCCGGATCTACAGGGGCATCCTTCGGTAAGTTAATTAATTTCTTTATCTTTAATTCAATTTTAATTACTTCAAATGGGTAGAGTTTTCTTTATT
TCAATCAGTTTGTTTTGCTATTGCAGACTAATGTATGAATTCTAATCAAAACGACATTTGATTTCAACTTATTCCGACAATCACATCCAGATTTCAGAAAAAATCTA
GTCGCAACAATAATTAATTTCAAGTTATGGACCTCCATTAACTTCATGTCCTCCCCCTTCCAAGCTCAAAATTTTAAGGCGAAACAAGAATATCATATATATATGA

ATTAATATGGTTTAACAAACATTAATAATCATAAAAATTACTTTAGATTGATTTTATTAATCTTTATTTTGTTTTATGATCAAAAGTGGATCCCAAACCTTATAACA
GATCCTAAACGACCTAATTATGAGCTATATACTACTCAGGTCCTGCTAAATAACTATTTTTTCAATTATGATTAAATACTTATTTTTGCTTTCTTATTCCCTAAAAT
TTACACTCTAACCTTTTTAAATGCAATTTCCTTTGTAAGAACAAATTCCATTCTTCATCTTCATCACTCATTAAACATTACACTTTGACCCTCCCACCAACCTTATG
AGTTCCAGCGTAATTTCTAGATTCCAACATTTATAGACAAAGAAGGATTATCGAGATTAAAGTGGTCAAATATAACGTTGTATTCCATTCAGATCATAATCCATTAG
ATTTTAAATTACAATATCATGAAGAGTAAATTAAAGTATCATTAATACAATTATTTGTTAAATATTACGTTGCACTTGTAGGACAAGAGATTGTGAATTATGAGAGC
CCGAGACCAACATTGGGGATTCACAGGTTTGTATTTGTGTTGTTTCGTCAACTGGGAAGACAAACTGTGTATGCACCAGCGTGGCGCCAGAATTTCATCACCAGAGA
CTTTGCTGAGCTTTACAATCTGGGATCTCCTGTGGCTGCTGTTTACTTCAATTGCCAGAAGGAGACTGGCTCTGGAGGAAGAAGAAGGCAGTAA

>Manin03g001820.1 (partial sequence).

TATCATGGCTAGGGAGAGTAGTATGAGAGATCCTCTTGCAGTCGGCAGAGTTATTGGAGACGTTCTTGATGAATTCACTAAGTCCGTTAGCCTCAAAGTGAGCTATA
GCAGCAAGGAAGTCAGCAATGGTGTTGAGCTCAAGCCCTCTCAAGTTGCAAATCAACCTAGGGTTGATATTGGTGGCACTGATCTCAGAGAATTCTACACTTTGGTT
ATTAACTTCCATCCCTTTTCTTCTTCCTCGTCTAGGGATTTTTTCTTCTAGGGTTTCTGGTTATGGTTGAATTTTTTTTAATCACATTTCAGGTGATGGTGGATCCT
GATGCTCCAAGCCCAAGTGTTCCCAGTTTGAGGGAGTACTTGCATTGGTAA

> Manin03g001830.1

ATTAATGATTTCATTAGCTTAAAAACCAATATAAATAGAGTTCCCATATGGACTTGGAAAACCAACAATATTTGGCTTGAATATTCTAACTACATCATGGATGGAGA
GGACTATGGTGAAAGTGCTTCCAGGGAGACTAGGGAAAGTGCTTCCAGAGATCCTCTTGTTGTTGGGCGAGTTGTGGGAGACGTTCTTGACAACTTTACTAAGTCTA
TTGCTTTCACTGCTAGCTATGGCCACAAGCTGGAAGTTCACAATGGTGTTTTACTCAAGCCCTCTCAAGTTGCCAAACAACCTAGGGTTTCTATTGGTGGAAGTGAT
TTCAGGAACTTCTACACTTTGGTAAGCCTCCTCTTCTCCAGCTATCCACCCTTTTCTTTAGCCCTATCTTCTTCATTTTTCCTGACCAAGTTTTTACACTAGAAAAG
ATTTATTTGCATCAGAAGTGTCCAGTCTTTTAACTATACTGATACACATGCATTTTATTTTTCGGTGCCTTGAATATTAATTAGCTCCATTGTAGGTCATGGTGGAT
CCTGATGCACCCAGTCCAAGTGACCCCCACCTTAAAGAGTACTTGCATTGGTGAATATTTTTTATGTTAAATTATATATATGTGTGTGTGTGTGTGTGTGTGTGTGT
GTGTGTGTGTGTGTGTGTATTATTCTTTTATATGGGAATTTATATCTAACTGGCTTTGAAAAAATGATAAATTCTGGTTAGGTTGGTGATTGATATTCCAGAAAGTA
CAGGAGCAACTTATGGTGAGTTGATTTTAACATTTTTGCAATCTATTTCAAATGGTTCTTAATGTGCTTGAGCTTACTTTAACTGTTAACTATAATCAATTGATTTT
TTTTCAAATTTCTAGTGCAAAGTGATGAATGAGTCACCTTTCATATGACATATTTGATTTGGATGTTCTGACTTGATTCTGCAGGAACTACTTGTGTTTTCAGTTCT
TAGACAAAAAGCAAAAGCTAATTTCAATGGACCTCCATAACTTGCCAATCATCTCATAGCTAAACTTTCTTCTTTATTTTCAAAAGGCAAATGACTATTTCCTACCC
GAGGTTTGATGCAAACCCAACTTTCCAACTACTAATTATCGAAAATCCAAATACCCACCTATCTTTTAAATTTTACTTTCATTGTTATTGTTTGTCATTTATTAAAA
TATTTATAAAAACTAAAAATTTATCATATTTCTCCCCAATGTTTAGTTTTCTAACAAATTTTCCCCCACCCAAAGTTTAAAAAATGAATATTTTCTCCTTAGGGTTT
TTCAGCCACCACTGTTAATGAATTATTTCTCTCTTTCTCTTTCGACTTCCCTCTCCATCTTAGTTCATCCCTGACCGTCATCGTATGATGAAAGTTATCAATGATAG
CATCTTTACTCATCCCAATGAAGTCGAATAGTTTTTGTCTCAAACGAAGGTAATGAATCATCATTGTATGAGAGACAATGATGATTCATCGCCTTCGTCTGATACAA
AGACAAAATATCGTCTTCGTCTAGATGAGCAGAAACATCATCGTTAGTCACTTTCATCGGACAATGATGGTCGGGGATGGACCAAGATGGAGAGAGAAACTAGGAGA
GAGAGAGAAATCAGTCATCATCGTCTCTAGCCGTCGGCAGTGATGGCTGGAAAACCTTAGGAGAGAAATATTCATTTTTCAAACATTGGGTGAGGAGGAATTTGTTA
GATTTTTAAAGTTAGGTGGGAAATTGTGATAAATTTTTAGTTTATTAAATATTTTAATAAATAACACTTTTACCCCTAATAGCAACAATAAAATTTCACAAATAGAT
GAGTATTTGAATTTTTAATAGTTAGCGGATGAAAATTTTGGTTTGTATTAAACCTTGGGTTTCAAAATAATATAATTTGCAACCCTAAATATTTAATCAGTAGAGCT
AGCCTCTTTTGAAATAATGCACCTAAATTTCAGTTTCTATCAAGGATCAAGGCATTAATTTCGGAGAAGGCAAGTTCAGAGTAATATAATATAAAAGAGAAAAAAAA
AATTAGGGTTAAGTATAAAAGCATTAAATGCAAACACAAACACAATGGTTAGAACAAAAGGCCAAAACCAAATTAATGAAAGAGCAATCTCAATTTTAAGCACCAAA
TTAGATTTTTAAATAATATATCCTTGAATGATTAAGTATTATTTTATATATAAATAATATGTTATTGTATGTTGTTAAAGGCAAAGAGGTTGTTTCTTATGAGAGCC
CAAAGCCAACAGTGGGGATTCACAGGTTTGTTTTTGTGCTGTTTCGGCAACCGGGGAAACAGCCTGTGTATGCACCAGGGTGGCGCCAGAACTTCGTAACCAGAGAC
TTTGCTGAGCTTTACAATCTTGGCTCCCCCGTGGCTGCCCTTTTTTTCAATTGCCAAAGGAGACTAAGACCTCTACTAGCTCATCATCAAGCTCAAGGAGACGCTA
G

C. *MiTFL1* genomic annotations and their corresponding genes from different mango cultivars.

MiTFL1 annotations from the mango genomic database (https://mangobase.org/easy_gdb/index.php), and their corresponding gene names from 'Alphonso' (2), 'SiJiMi' (4) and 'Shelly' (this study), cultivars.

	***MiTFL1-like* genes' genomic annotations**	**'Alphonso' cloned *TFL1* genes [2]**	**'SiJiMi' cloned *TFL1* genes [4]**	**'Shelly' cloned *TFL1* genes (this study)**
1	Manin19g005780.1	*MiTFLα* (KU206290)	*MiTFL1-1*#	*MiTFL1-1* (OP341602)
2	Chr 3 unannotated	*MiTFL1* (KF258590.1)	*MiTFL1-2* #	*MiTFL1-2* (OP341603)
3	Not found		*MiTFL1-3*#	
4	Manin01g011190.1		*MiTFL1-4*#	

Accession numbers not assigned.

(4). Wang, Y.-H.; He, X.-H.; Yu, H.-X.; Mo, X.; Fan, Y.; Fan, Z.-Y.; Xie, X.-J.; Liu, Y.; Luo, C. Overexpression of four MiTFL1 genes from mango delays the flowering time in transgenic Arabidopsis. BMC Plant Biol. 2021, 21, 1–16. doi:10.1186/s12870-021-03199-9.

> Manin19g005780.1

ATGGCAAGAATAGTAGACCCTCTTGTTGTGGGGAGAGTGATAGGAGATGTTCTTGATTCTTTCTCTCCAACAATCACAATGTTTGTAAGTTACAATAACAGGCAAGT
CTGTAATGGCCATGAGCTTTTACCTTCCACAGTTTCCTTCAGACCTAGGGTTGAGATTCAAGGAGGTGATATGAGAACTTTCTTTACACTGGTATCCTCCATTCTTT
TACTCTTTTTAGATTTCCCTTCTACAATCACTCATTTTGCATCTTGCTAACAAAATAAACTAATTTTTGCCTCGTTAATTGCAGGTGATGACAGACCCAGATGTTCC
TGGACCTAGTGATCCTTACTTAAGGGAGCACCTGCACTGGTATACTCTAAAACCATCAAAAAAAAAAAAAAAATCTGGTGAATATTAGGATACTCTTATCATGAGCC
TTCTATTATTGAACCTATCAGTGTTAGTACAAACATGTGTCAAAGTGAAAACCCTAGTTCATGGTTGATCACCAACTTTCTTCAAAACAAGAAAAACTAGTTTCACC
AAAAGCCAAAGGGAACATCTATAGAAATGGCATTTATTTTATGTACATAGAATCCCATCTGCAGACATCAACCATACTTTTAATCAATACATTATTGTCACATAATT
TTTTAGACTCAATACCTATTATCCATTTAAGTAGAGAGAAAATACTTACATGTTCTGTAAATTACAGTATTCAAGTTACTACTTTTTCAAACCCTGTAGCCAAGTTG
AAGCAAATGCATGCTCAAAGCTCAATCATACCAAGATAATTTAATCTACAGCTGCTAAGTTAAAACTAACAATAATTCTGGGTTAATCTGTTTACAGGCTAGTGGCA
AACATACCAGGAACAACAGATGTCACATTTGGTAGGTCATATGATTTACTTTTGTAGATCTTAAAGGATTTTAACAGTTTTCATTGAGGGATTTCATGTATGTCAGG
GAGGGAACTGGTGAGTTATGAGATACCAAGGCCAAATATAGGCATCCACAGATTTGTTTTTGCTCTATTCAGGCAGAAAGGTAGACAGATATTTAACCCACCTTCTT
CAAGGGATAACTTCAGCACTCGAATTTTCGCTGCTGAAAACGATCTCGGTCCTCCTGTTGCTGCCGTCTACTTCAACGCACAAAGAGAAACTGCAGCTAGAAGACGC
TAA

> Chromosome 3 Transcript from 17065640- 17066894 on minus strand.

GAAATCATTATGCTTGATTGATGGCTCAAATGTAATACATAAAATTCCGTGTATAATCTTCTTGTTTCTCATGCAAATGGCATATCTTTTAATTTAATTTCGTGGAA
CAACTAAAAATCTGCAAATAAGTTCTCCAAAATTTATGTTTTCAGCTGATTACTTTTGTAAAAAAATTTATAAATTTTATCATATAATTACAATATATATAGGACCC
ACAATATACTCAGTCCAATAACTTTGACACCTATTTAATCTAATCAATCATTTTAATCAGATGGAGTGCTTCCCCATTCAATTACTACTGAATCATTGATATATTAT
ATTTGTAACTTATCTAATAGTGAGATCAACAACTTTTTGTTTACAGGAATCAAATAAATAAATACCCAACTGTTAATTTGACCTGACTTTTGCTATGACATTCAAAG
TATATTGTGTTTCAAGATGATGAATTTCGTTGGCTTCAAACTTTGAACAAATTTAGTTTATGACTTTCTAAGTATATTTTGTCTCACGATGATGAATTTCATTGGCT
TCAAACTTTGAACAATTTAGTTTTTGTTTGTAGTAATGGTAAAAAGACAACATTCGTGTCGCAATTTGGGAGTTCAGAATTAATTTAAGTAATGCATTGATTTGATT
AAAAGATTACGTGCGACGTGTTAGGAAGAATAAGAATCTAATTCTTAACCCTATGATTTCTCTCAGGCTACTATATGCCCTGTAATTCATATTTAAAACATGACACC
ATTTATTTTTATCAATGTCCCAGGTTAATTTGGACATTTTGGCAATCCAAACCAAGCATTTGGCCTAGGTTCCATGGATCTAACTACCTTCTCTACACTATAAATAG
GGAGCTTCAACTTCAAGGGAAGATAAGTAAAAGAAAGAAAAACACATAATTAGATCATTCTCTCTCTCCAATGGCAAGAATGCCATCAGATCCTCTTGTAGTTGGCA
GAGTGATTGGAGATGTTGTTGATTGTTGTGATCAAACTGTCAAAATGGCAGTCACCTACAACTCTTCCAAGCAGGTGTACAATGGCCATGAGCTGTTTCCATCTTCA
GTGACTGTGAAACCTAAGGTTGAGGTTCACGGAGGTGATATGAGATCATTCTTCACATTGgtaaactttactacttattcttcttaattttctcttctttttttata
tcgtctgcttcatggagttaattaaagagtcttcttctttactataatagATCATGACAGACCCAGATGTTCCTGGTCCAAGTGACCCATACTTGAGGGAGCATTTA
CACTGgtataatctcaaactctcccccaatattatatgctgattaattacaagaaattaaccaaaagaatgaacaggtttcagttatgcccctatgtcatagcacat
gtaaaacaactatccatagcacatgcatgtataaactttgctcaaattaatcaaccaatcgtgacctaatttttgaaatgcttcaagagttattgctgtgtatgcat
cgtcaatatgatatatttcaagaaggattcattttgcattttaatttcttcaatatatggaagaaaaagtttgtgtatatcacatgtgattgtatcaatgcaaaatc
tctcaactttaattgagtatccttacagGGTGGTGACAGACATCCCAGGCACAAcTGATGCCACGTTTGgtaagtagtttcataatttagagaaaaagacagagtaa

taaattttgatacaggaaaggttaatatgtatttatgtgttaaattacagGAAGGGAATTGGTGAACTATGAAATGCCGAGGCCAAATATTGGGATTCACAGGTTTG
TTTTCCTTCTGTTCAGGCAGAAACGCAGGCAAACAGTGATAAGTATACCATCTTCAAGGGACGGCTTCAACACAAGAAAGTTTGCTGAAGACAATGACCTTGGTCAG
CCTGTTGCAGCTGTGTTCTTCAATGCACAAAGGGAAACAGCCGCAAGAAGGCGCTGATAAATCAGGAAATAATTATAAAAAATAAGGTACTATTCCAGAAGGAAAAA
ATATTTAAAACATTTTAATTTGAAAAGGCGGTGGGTGCAGTAGTTTTATATAGTGTTTAGTGGGTCATCAAGAATAAATCCTTGGTAAATAAGTATGTACTATGCAT
GTCATATCTCTGAGTGGATATATATGTAACCTATCCCAAAGTACCAGGTTTTCAAGTTACTTATAAAATGAATCATTTGATGGCTTAAGAGTGTTACTTTGTAGTTT
GATTTTCTAGAAATAAAATGGAAGAAATAATGGTATATTGGTTAATGGGGGTTCCAATAGTTAAATCCCACTTCGTTTTCCATACCTCAAATCTCACGTTCACGTTA
CTTAAGCAGATGAATGTCGCTTATCGTTCTTTGCGTGTTTATTTGACCATGACTTCTGGGGCTTTCCTTAAGGTTTTTTTTCTTTTTGGGTAATTAGCTTTCCTGAA
GTTTCTTTGGGTAATTTTTTCCTTTCCTTTCTTTTATGATTTGACAACGATGACTACAGACAGTGTAAATTTTCAGAATCACCTGTTTTCACCTACAAAGAGTACCC
TTCCATTTTCCATTTTCATCTCGATTTCTCTGCACTTTGGCAGTAAATATTAGTAAGAATGCTTCTCTTTTTGTATCACCTTTTTCCAGGTAAAAAAGAGTTTCAAT
TTTCTGCAGTAGCATAATCATAGATACCTTGAGATCAGAATGAAAACACCAGTTGCCTATTTTCGAGGCTAGTGCCAAAAGAACCTTACTGGCCTGTAGGGTGACTT
CAAAAAGTGTGGGAAGGCCAAAGTTTCCAAAGGCGTGCACAGCACCCGATTAAAGAAATATAGGAAAATTATAAATGCTCAAAGAAGTAAAACTTACTTGCTACTTC
ATCCACCTTTAATGCTAGAAATATTAATTTTGTAATTAAAAATCCTATTTTTTTAAATAAAAATGTTCTTAAAATGACATTAACTTAACATTACTGAAAAAAATAAT
ATTTATATAAAATTACATTTTGTCTTTACATAGTTTAGTGCATATTAGAATCATGAAAATCTATTTCAATAATTTAAAGGCCAAAAGACTTATTCCCACCCAAAGTA
TATTGTATTCCCAAGTTTTTATCATTTAACTTTGAAATTTTCATTTACCTATTTATGGA

>Manin01g011190.1

AGCTTCCTCTTAAGCTTTCCTTGAGAGTTGATGAATGGCGGAGCCCCTGGCTGTAGGGAGAGTGGTGGGTGATGTTGTGGACAATTTCAGCCCCAGTGTGAAGATGA
CTGTGACTTATAGCTCCAATAAGCAAGTCGCTAATGGTTATGAGCTTATGCCTGCTGCCACTGCTGCTAAACCTCGGGTTGAGATTGGTGGCGAGGACTTGAGAGCT
GCCTACACATTGGTAAATATAAACTTTCATATATGTGTGTGTGTATATCTATATATATGTATCATAATTTGAACACGCAATAATAAAAATAATGTTATGTGCATCCA
ATTTTGAGTACCCAATTAGATATTTAAATAACGTGTCATTATATAATTAGAATGATAAGACATATGAATATTCAATTAAGTTCTTAAAATTGTATACATATAATTTT
ACTATAATTATAATGCTAAGTCCAGTACTAACAACTATCTGCTTTTTTTTTTTGCGTGTCATCTCTTTGTTGAGTAGATCATGACTGACCCTGATGCTCCAAGCCCT
AGTGATCCATGCATGAGAGAACATCTCCACTGGTTAGTATATAATATATATATATATATATATATATATATATATATATACAAAGACTCATGTTTAACCTTCACA
TGGGACAAATAATATGAGAGACGTTATTTGATTAGTTCTGAGAATGTATAAGGTAGTAAAGGATTCCTCTAGCTACCAGCATAAGATTTTTACTCTGATAGAACCTT
GTGCACATGTGGTTTCAGAGCCACTAGGGTTCAAGTCCCATCAAAGATTGTAGTGACTTCTCACATGGGACAACCAACATGAGAGATACCACCTACCTGATTGGTTC
TAAGGACATACGAGGCATCAAGGGCTAAGTAAAGCATGAAAGCTTAAGCTGTTAGCTCTGATAGTACCTTGTTATTTAACATTTCATTGTACATTAATTTGTTTCAT
AGCAATCAAATTGTCTCACAGAACTTTTTCACTTTCAGGATGGTTACAGACATTCCTGGAACAACTGATGTTTCCTTTGGTTAGTTTTGGAGCTTGAAAATAATACA
CTCCGTTTGAATTTAAGAGATCATTTGAAGAACTTAACTGGGTTGTTTTAATTTGAATGGTAATGTAAAGCAGGAAAAGAAGCGGTGGGTTATGAGATTCCAAAGCC
CGTGGTGGGCATCCATAGATACGTATTCATATTGTTCAAACAAAGAGGAAGACAGACTGTGAAGGCACCAACTTCGAGGGACCATTTCAACACAAGACAGTTTGCAC
AGGACAACGGCCTTGGCCTGCCTGTGGCTGCGGTCTACTTTAATGCCCAGAGAGAAACTGCTGCAAGAAGAAGATGAAGAAAAAAGAAAGAGTTCAATACTGCTTCA
GGCGAAATAAATAAAACTGCCAACTTCAACTTGAATTTGGCAGCTCTAGTTCTTGTTTTAGGGTCTTGGGTTCAAATGTCTGTTTGTAGAAGCACTATTTTCCTCTG
CTTGATGCGTTTGATTAGTTTATTATTAACATGTTCTGATTTACCGTAAAATTAGCTTCTTCTATGAACAGGATCATATAC
