# Supplementary material for: Expression Profiling of Four Mango FT/TFL1-Encoding Genes under Different Fruit Load Conditions, and Their Involvement in Flowering Regulation
Source: Plants (Basel). 2022 Sep 15;11(18):2409. doi: 10.3390/plants11182409 (PMC9506463; doi:10.3390/plants11182409)
Supplement: Supplementary file 1 [file plants-11-02409-s001.zip › Sup Tables Gafni et al FINAL.pdf]

Table S1. Primers used for *MiFT/MiTFL1* gene isolation, real-time qPCR analysis and plasmid preparation.

Gene name	Primer name	Primer sequence 5'→3'	Direction	Annealing temperature
		**Primers used for *MiFT/MiTFL1* gene isolation**		
MiFT1	MiFT1-1	5' TTTACTATGTCTTGGGAAGGCT 3'	S	58
	MiFT1-2	5' ACATAAGCATATTACTGCCTTCTTC 3'	AS	61
MiFT2	MiFT2-1	5' CATCATGGATGGAGAGGACTATG 3'	S	63
	MiFT2-2	5' GAAGTCTCTACATGACAGCCTATAA 3'	AS	63
MiTFL1-1	MiTFL1-1-1	5' CTCCTACTGATTCAATGGCAAGAA3'	S	61
	MiTFL1-1-2	5' GGTTTCCAAATAGACTGGACGA 3'	AS	60
MiTFL1-2	MiTFL1-2-1	5' GTTCCATGGATCTAACTACCTTCTC 3'	S	64
	MiTFL1-2-2	5' CTGCACCCATCGCCTTT 3'	AS	55
		**Primers used for *MiFT/MiTFL1* gene real-time qPCR**		
MiFT1	MiFT1-3	5' GGTTGGTGACTGATATTCCC 3'	S	58
	MiFT1-4	5' GTTTGTCTTCCCAGTTGACG 3'	AS	58
MiFT2	MiFT2-3	5' TCAAGGAGACGCCAGCTAAT 3'	S	58
	MiFT2-4	5' GAAGTCTCTACATGACAGCCTAT 3'	AS	61
MiTFL1-1	MiTFL1-1-3	5' GGTAGACAGATATTTAACCCACCTT 3'	S	63
	MiTFL1-1-4	5' AGGAGGACCGAGATCGTTT 3'	AS	57
MiTFL1-2	MiTFL1-2-3	5' CGCAGGCAAACAGTGATAAGTAT3'	S	62
	MiTFL1-2-4	5' CCTTTGTGCATTGAAGAACACA3'	AS	62
MiAP1	MiAP1-1	5' AGAACAAGATCAACCGTCAGGTC 3'	S	65
	MiAP1-2	5' GCAACTTCAGCATCACAAAGAAC 3'	AS	64
MiEF	MiEF-1	5' CATTGTGGTCATCGGCCATGTCG 3'	S	65
	MiEF-2	5' TCACACGCTTGTCAATACCTCCAAG 3'	AS	64
		**Primers used for plasmid preparation**		
MiFT1	MiFT1_EcoRI	5' AAGTGAATTCTTTACTATGTCTTGGGAAGGCT 3'	S	57
	MiFT1_XbaI	5' TTCATCTAGAACATAAGCATATTACTGCCTTCT 3'	AS	57
MiFT2	MiFT2_EcoRI	5' AAGTGAATTCCATCATGGATGGAGAGGACTATG 3'	S	60
	MiFT2_XbaI	5' TCATCTAGAGAAGTCTCTACATGACAGCCTATAA 3'	AS	60
MiTFL1-1	MiTFL1-1_EcoRI	5' AAGTGAATTCCTCCTACTGATTCAATGGCAAGA 3'	S	58
	MiTFL1-1_BamHI	5' TTCTGGATCC GGTTTCCAAATAGACTGGACGA 3'	AS	58
MiTFL1-2	MiTFL1-2_EcoRI	5' AAGTGAATTCGTTCCATGGCAAGAATGCCATCA 3'	S	58
	MiTFL1-2_BamHI	5' TTCTGGATCCCTGCACCCATCGCCTTT 3'	AS	58

Table S2. R^2 values and PCR efficiency obtained for the standard curves of the examined genes.

Genes	R^2	Efficiency
MiEF	0.999	105.01
MiFT1	0.997	95.46
MiFT2	0.994	103.30
MiTFL1-1	0.992	94.22
MiTFL1-2	0.991	101.54
MiAP1	0.999	96.15
