# Supplementary material for: Expression Profiling of Four Mango FT/TFL1-Encoding Genes under Different Fruit Load Conditions, and Their Involvement in Flowering Regulation
Source: Plants (Basel). 2022 Sep 15;11(18):2409. doi: 10.3390/plants11182409 (PMC9506463; doi:10.3390/plants11182409)
Supplement: Supplementary file 1 [file plants-11-02409-s001.zip › Suppl figures Gafni et al FINAL.pdf]

```
MiFT1-Shelly        MSWEGCCSDRDPLVVGRVIGDVIDNFNTSISRNVSYGNRDVGNGVELKPSVVANHPRVDI   60
MiFT-Irwin          MSWEGCCSDRDPLVVGRVIGDVIDNFNTSISRNVSYGNRDVGNGVELKPSVVANHPRVDI   60
MiFT1-Alphonso      MSWEGCCSDRDPLVVGRVIGDVIDNFNTSISCNVSYGNRDVGNGVELKPSVVANHPRVDI   60
MiFT1-SiJiMi        MSWEGSCSNRDPLVVGRVIGDVINNFNRSVSLNVSYGNRDVNNGVELKPAVVANHPRVDI   60
                    *****.**:**************:*** *:* *********.*******:**********

MiFT1-Shelly        GGTDLRTFYTLVMVDPDAPSPSNPGLREYLHWLVTDIPGSTGASFGQEIVNYESPRPTLG   120
MiFT-Irwin          GGTDLRTFYTLVMVDPDAPSPSNPSLREYLHWLVTDIPGSTGASFGQEIVNYESPRPTLG   120
MiFT1-Alphonso      GGTDLRTFYPLVMMDPDAPSPSNPSLREYLHWLVTDIPGSTGASFGQEIVNYESPRPTLG   120
MiFT1-SiJiMi        GGTDLRTFYTLVMVDPDAPSPSNPSLRFYLHWLVTDIPGSTGASFGQEIVNYESPRPTIG   120
                    *********.***:**********.** ******************************:*

MiFT1-Shelly        IHRFVFMLFRQLGRQTVYAPAWRQNFITRDFAELYNLGSPAAAVYFNCQKETGSGGRRRQ   180
MiFT-Irwin          IHRFVFMLFRQLGRQTVYAPAWRQNFITRDFAELYNLGSPVAAVYFNCQKETGSGGRRRQ   180
MiFT1-Alphonso      IHRFVFMLFRQLGRQTVYAPAWRQNFITRDFAELYNLGSPVAAVYFNCQKETGSGGRRRQ   180
MiFT1-SiJiMi        IHRFVFVLFRQLGRQTVYAPAWRQNFITRDFAELYNLGSPVAAVYFNCQKETGSGGRRR    179
                    ******:*********************************.******************
```

Figure S1. (A) Amino acid sequence comparison and structural features of mango cv. Shelly *Mi*FT1 (accession number: OP341600). Comparison of deduced amino acid sequence of *MiFT1* from 'Shelly' (this study) to *Mi*FT from 'Irwin' (accession number [ac]: AB671587.1), *Mi*FT1 from 'Alphonso' (ac: JX177434.1), and *Mi*FT1from 'SiJiMi' (ac: MT419778). The two conserved amino acids Tyr-89 and Gln-144 (corresponding to Tyr-85 and Gln-140 in *At*FT), required for FT activity, are highlighted in purple. The conserved amino acid sequence in *At*FT1 C-terminal region, with Ala-141 corresponding to Gly-137 in *At*FT, is highlighted in yellow. The three conserved amino acids Val-74, Ser-80 and Arg-87 (corresponding to Val-70, Ser-76 and Arg-83 in *At*FT), which are suggested to play an essential role in regulating AtFT transport, are highlighted in green.

```
MiFT2-Shelly         MDGEDYGESASRETRESASRDPLVVGRVVGDVLDNFTKSIAFTASYGHKLEVHNGVLLKP   60
MiFT-like-Keitt/TA   MDGEDYGESASRETRESASRDPLVVGRVVGDVLDNFTKSIAFTASYGHKLEVHNGVLLKP   60
MiFT3-SiJiMi         MDGEGYGESASRETRESASRDPLVVGRVVGDVLDNFTKSIAFTASYGHKLEVHNGVLLKP   60
                     ****.*******************************************************

MiFT2-Shelly         SQVAKQPRVSIGGSDFRNFYTLVMVDPDAPSPSDPHLKEYLHWLVIDIPESTGATYGKEV   120
MiFT-like-Keitt/TA   SQVAKQPRVSIGGSDFRNFYTLVMVDPDAPSPSDPHLKEYLHWLVIDIPESTGATYGKEV   120
MiFT3-SiJiMi         SQVAKQPRVSIGGSDFRNFYTLVMVDPDAPSPSDPHLKEYLHWLVIDIPESTGATYGKEV   120
                     ************************************************************

MiFT2-Shelly         VSYESPKPTVGIHRFVFVLFRQPGKQPVYAPGWRQNFVTRDFAELYNLGSPVAALFFNCQ   180
MiFT-like-Keitt/TA   VSYESPKPTVGIHRFVFVLFRQPGKQPVYAPGWRQNFVTRDFAELYNLGSPVAALFFNCQ   180
MiFT3-SiJiMi         VSYESPKPTVGIHRFVFVLFRQPGKQPVYAPGWRQNFVTRDFAELYNLGSPVAALFFNCQ   180
                     ************************************************************

MiFT2-Shelly         KETKTSTSSSSSSRRR                                               197
MiFT-like-Keitt/TA   KETKTSTSSSSSSRRR                                               197
MiFT3-SiJiMi         KETKTSTSSSSSSRRR                                               197
                     ****************
```

Figure S1. (B) Amino acid sequence comparison and structural features of 'Shelly' *Mi*FT2 (accession number: OP341601). Comparison of deduced amino acid sequence of *MiFT2* from 'Shelly' (this study) to *Mi*FT-like from 'Keitt' and 'Tommy Atkins'(Keitt/TA) (transcriptome data base; mango_rep_c5502) and *Mi*FT3 from 'SiJiMi' (ac: JQ700254.1). The two conserved amino acids Tyr-89 and Gln-144 (corresponding to Tyr-85 and Gln-140 in *At*FT), required for *At*FT activity, are highlighted in purple. The semi-conserved amino acid sequence, found in the C-terminal region of the proteins, with Pro-143, Lys-145 and Pro-147 (highlighted in gray) instead of Leu-128, Arg-130 and Thr-132 in *At*FT, is highlighted in yellow. Two out of three conserved amino acids: Val-74 and Ser-80 (corresponding to Val-70 and Ser-76 in *At*FT), which are suggested to play an essential role in regulating FT transport, are highlighted in green.

```
MiTFL1-1-Shelly      MARIVDPLVVGRVIGDVLDSFSPTITMFVSYNNRQVCNGHELLPSTVSFRPRVEIQGGDM    60
MiTFLα-Alphonso      MARIVDPLVVGRVIGDVLDSFSPTITMFVSYNNRQVCNGHELLPSTVSFRPRVEIQGGDM    60
MiTFL1-1-SiJiMi      MARIVDPLVVGRVIGDVLDSFSPTITMFVSYNNRQVCNGHELLPSTVSFRPRVEIQGGDM    60
                     ************************************************************

MiTFL1-1-Shelly      RTFFTLVMTDPDVPGPSDPYLREHLHWLVANIPGTTDVTFGRELVSYEIPRPNIGIHRFV    120
MiTFLα-Alphonso      RTFFTLVMTDPDVPGPSDPYLREHLHWPVANIPGTTDVTFGRELVSYEIPRPNIGIHRFV    120
MiTFL1-1-SiJiMi      RTFFTLVMTDPDVPGPSDPYLREHLHWLVANIPGTTDVTFGRELVSYEIPRPNIGIHRFA    120
                     *************************** *******************************.

MiTFL1-1-Shelly      FALFRQKGRQIFNPPSSRDNFSTRIFAAENDLGPPVAAVYFNAQRETAARRR            172
MiTFLα-Alphonso      FALFRQKGRQIFNPPSSRDNFSTRIFAAENDLGPPVAAVYFNAQRETAARRR            172
MiTFL1-1-SiJiMi      FALFRQKGRQIFNPPSSRDNFSTRIFAAENDLGPPVAAVYFNAQRETAARR             171
                     ***************************************************
```

Figure S1. (C) Amino acid sequence comparison and structural features of 'Shelly' *Mi*TFL1-1 (accession number: OP341602). Comparison of deduced amino acid sequence of *MiTFL1-1* from 'Shelly' (this study) to *Mi*TFLα from 'Alphonso' (ac: AND81244.1) and *Mi*TFL1-1 from 'SiJiMi' (Wang et al., 2021). The two crucial conserved amino acid residues His-85 and Asp-140 (corresponding to *At*TFL1), which are required for TFL1 anti-florigenic function, are highlighted in purple.

```
MiTFL1-2-Shelly      MARMPSDPLVVGRVIGDVVDCCDQTVKMAVTYNSSKQVYNGHELFPSSVTVKPKVEVHGG   60
MiTFL1-2-SiJiMi      MARMPSDPLVVGRVIGDVVDCCDQTVKMAVTYNSSKQVYNGHELFPSSVTVKPKVEVHGG   60
MiTFL1-Alphonso      MARMPSDPLVVGRVIGDVVDCCDQTVKMAVTYNSSKQVYNGHELFPSSVTVKPKVEVHGG   60
                     ************************************************************

MiTFL1-2-Shelly      DMRSFFTLIMTDPDVPGPSDPYLREHLHWVVTDIPGTTDATFGRELVNYEMPRPNIGIHR   120
MiTFL1-2-SiJiMi      DMRSFFTLIMTDPDVPGPSDPYLREHLHWVVTDIPGTTDATFGRELVNYEMPRPNIGIHR   120
MiTFL1-Alphonso      DMRSFFTLIMTDPDVPGPSDPYLREHLHWVVTDIPGTTDATFGKELVNYEMPRPNIGIHR   120
                     *******************************************:****************

MiTFL1-2-Shelly      FVFLLFRQKRRQTVISIPSSRDGFNTRKFAEDNDLGQPVAAVFFNAQRETAARRR       176
MiTFL1-2-SiJiMi      FVFLLFRQKRRQTVISIPSSRDGFNTRKFAEDNDLGQPVAAVFFNAQRETAARR        176
MiTFL1-Alphonso      FVFLLFRQKRRQTVISIPSSRDGFNTRKFAEDNDLGQPVAAVFFNAQRETAARRR       175
                    ******************************************************
```

Figure S1. (D) Amino acid sequence comparison and structural features of 'Shelly' *Mi*TFL1-2 (accession number: OP341603). Comparison of deduced amino acid sequence of *MiTFL1-2* from 'Shelly' (this study) to *Mi*TFL1-2 from 'SiJiMi' (Wang et al., 2021) and *Mi*TFL1 from 'Alphonso' (ac: AGW23412.1). The two crucial conserved amino acid residues His-85 and Asp-140 (corresponding to *At*TFL1), which are required for TFL1 anti-florigenic function, are highlighted in purple.

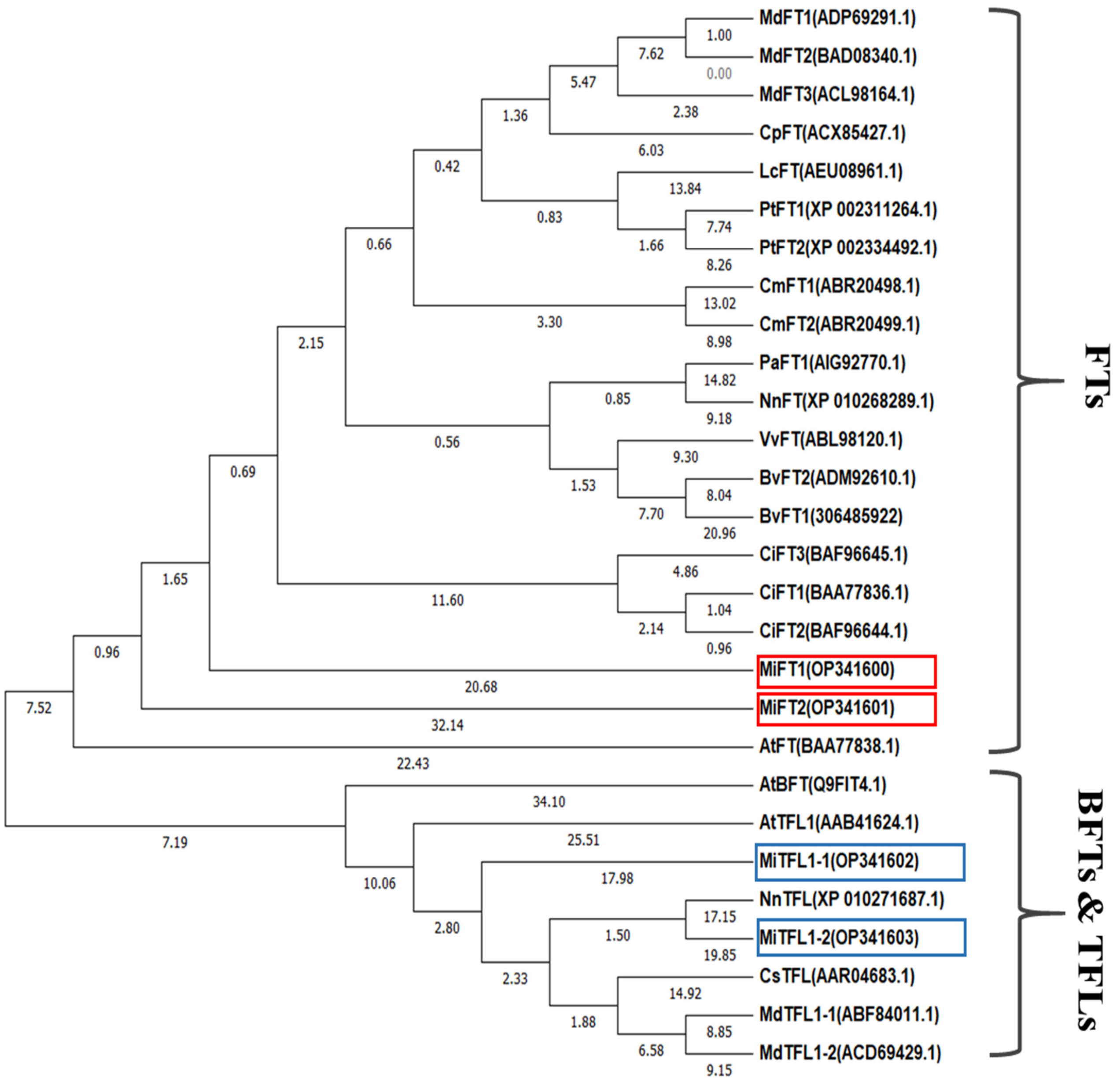

Figure S2. Phylogeny of FT, TFL and BFT proteins. Conserved amino acid sequences of MiFT/TFL1 proteins (boxed) and FT, TFL and BFT proteins from various plant species (Vv, *Vitis vinifera*; At, *Arabidopsis thaliana*; Pt, *Populus trichocarpa*; Mi, *Mangifera Indica*; Cs, *Citrus sinensis*; Md, *Malus domestica*; Nn, *Nelumbo nucifera*; Ci, *Citrus unshiu*; Cm, *Cucurbita moschata*; Lc, *Litchi chinensis*; Cp, *Carica papaya*; Pa, *Persea Americana*; Bv, *Beta vulgaris*). Database accession numbers are given. Bootstrap consensus trees were inferred from 1000 replicates and graphically designed with iTOL (Interactive Tree of Life, V5). Branch lengths are indicated.

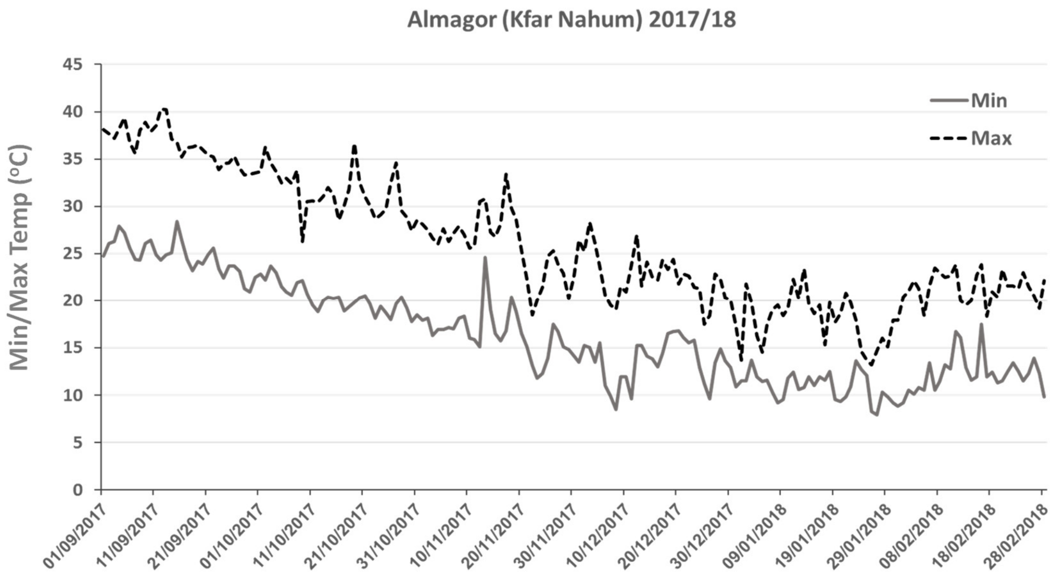

Figure S3. Minimal and maximal temperatures recorded from September 2017 through February 2018 at Kfar Nahum near Almagor, where experimental 'Shelly' trees were grown.
